# Metagenomic sequencing reveals structural and functional differentiation of rhizosphere bacterial communities driven by nitrogen and potassium deficiency associated with root rot of *Schisandra chinensis*

**DOI:** 10.3389/fmicb.2026.1827096

**Published:** 2026-05-13

**Authors:** Li Li, Ruibo Liu, Hao Yang, Yongping Zhao

**Affiliations:** 1 College of Biology Pharmacy and Food Engineering, Shangluo University, Shangluo, Shaanxi, China; 2 College of Agriculture, Shanxi Agricultural University, Taigu, Shanxi, China

**Keywords:** bacterial communities, functional differentiation, metagenomic sequencing, *Schisandra chinensis*, structural

## Abstract

**Background:**

Frequent incidence of root rot in *Schisandra chinensis* impairs its yield and quality, yet the rhizosphere microecological mechanism driving this incidence remains unclear.

**Methods:**

To clarify this mechanism, healthy and root rot-infected *S. chinensis* plants were analyzed in this study. The plant growth, rhizosphere soil physicochemical properties, and the structural and functional differences in rhizosphere bacterial communities under both conditions were analyzed.

**Results:**

Our results showed that root rot significantly inhibited *S. chinensis* growth and pathogen colonization-induced rhizosphere acidification, with reduced hydrolyzable nitrogen (HN) and available potassium (AK). Analysis of the intergroup differences in bacterial species revealed that the healthy rhizosphere was enriched with Acidobacteriota, *Luteitalea*, Pseudomonadota, *Pseudolabrys*, and Methylomirabilota, whereas infected rhizosphere was dominated by *Gaiella* (Actinomycetota), *Gemmatimonas* (Gemmatimonadota), *Bradyrhizobium*, and *Sphingomicrobium* (Pseudomonadota). Functional annotation based on COG, KEGG, and CAZy databases revealed that the bacteria of the healthy rhizosphere were enriched in defensive-cooperative functions (synergistic metabolism, secondary metabolite synthesis, complex carbon metabolism), while those of the infected rhizosphere exhibited simplified survival functions (individual metabolism, ABC transport, simple carbohydrate metabolism). Redundancy analysis identified HN and AK as key nutrients driving community differentiation in the rhizosphere.

**Conclusion:**

This study revealed that root rot in *S. chinensis* is closely associated with an imbalance in the rhizosphere environment–bacterial community–function system, with healthy plants exhibiting specific core bacterial biomarkers and more complex synergistic metabolic networks, while HN and AK are key nutrients influencing rhizosphere bacterial communities. This study clarifies the rhizosphere microecological mechanism associated with *S. chinensis* root rot, providing a theoretical basis for its control.

## 1 Introduction

*Schisandra chinensis* (Turcz.) Baill. is a deciduous woody vine belonging to the family Schisandraceae. Its name comes from the five distinct flavors (sweet, sour, bitter, pungent, and salty) of its flesh, pulp, and seeds (Rybnikář et al., 2019). It is highly valued for its medicinal properties and is widely distributed across East Asia, including China, the Korean Peninsula, the Russian Far East, and Japan. Shangluo in Shaanxi Province is recognized as one of its genuine producing areas and has witnessed a continuous expansion of cultivation in recent years. Thus, *S. chinensis* serves as a pivotal pillar of the medicinal plant industry of Shangluo City. The pharmacological value of this traditional, authentic Chinese medicinal herb, *S. chinensis*, has been extensively acknowledged, with research focused on the clinical applications of bioactive compounds in its fruit. However, systematic research on the rhizosphere microecology is relatively less.

The rhizosphere is a crucial interface between plant roots and soil microorganisms. Specifically, the bacteria of the rhizosphere are the most metabolically active functional units in soil ecosystems, and they participate in nutrient cycling through metabolic turnover, regulate the biosynthesis of plant bioactive components (Zhang et al., 2025). Soil microorganisms enhance plant growth and stress resistance by improving soil properties and activating nutrients (Ling et al., 2022; Wang F. et al., 2022), boost disease resistance by inducing systemic resistance (ISR) and exerting antagonistic effects against pathogens (Beneduzi et al., 2012; Santoyo, 2022), and alleviate the toxicity of pesticides and heavy metals via biodegradation, chelation, and ROS scavenging (Wang G. et al., 2022). Studies have confirmed that the characteristics of the microbial community in the rhizosphere of medicinal plants are closely related to the host’s health and quality. For instance, Candidatus Solibacter and Bryobacter genera in the rhizosphere of *S. chinensis* are positively correlated with the content of schisandrol A (Wang et al., 2024). Similarly, Planctomycetota in the rhizosphere of *Atractylodes macrocephala* directly influence the accumulation of atractylenolide (Bai et al., 2025a). Concurrently, alterations in soil physicochemical properties reshape the rhizosphere microbial flora and indirectly affect plant growth. Research has also demonstrated that the fluctuations in pH and nitrogen content of the rhizosphere of *Panax quinquefolius* disrupt the bacterial community structure and thereby induce growth disorders (Liu et al., 2021). Therefore, maintaining the stability of the rhizosphere bacterial community and the balance of soil physicochemical properties is crucial for the healthy growth of medicinal plants (Yang et al., 2021; Yu T. et al., 2022). However, research on the rhizobacterial community structure and soil physicochemical properties of *S. chinensis* remains scarce.

With the intensification of artificial cultivation of *S. chinensis*, root rot has emerged as a major constraint for industrial development. This soil-borne disease causes plant wilting and death (Bischoff Nunes and Goodwin, 2022) and significantly reduces fruit quality and yield. Currently, the microecological mechanisms underlying the occurrence of root rot in *S. chinensis* remain unclear. To address this knowledge gap, the present study analyzed the rhizosphere soil samples from the healthy and root rot-infected *S. chinensis* plants in the Guowan Village (Shangzhou District, Shangluo City, Shaanxi Province). The structural and functional differences in the bacterial community between the two samples were analyzed based on metagenomic sequencing. Furthermore, the intrinsic correlations among plant growth, soil physicochemical properties, and bacterial community traits were analyzed to identify the key soil factors driving changes in the structure and function of the rhizobacterial community of *S. chinensis* under root rot stress. The study’s findings will provide novel insights into the rhizosphere microecological mechanisms governing the occurrence of root rot in *S. chinensis* and a scientific basis for the cultivation and management of this valuable medicinal plant.

## 2 Materials and methods

### 2.1 Overview of the sampling site

The site from where the rhizosphere samples were collected for this study was located at the *S. chinensis* Base in Guowan Village, Beikuanping Town (Shangzhou District, Shangluo City, Shaanxi Province; 110°15′N, 33°32′E) (Supplementary Figure S1). The base, established in 2020, has a planted area of 72 mu (1 mu ≈ 666.7 m^2^) and a planting density of 1,500 plants per mu. The experimental area has a mild and humid climate, with an annual average precipitation of 971 mm, an annual average temperature of 12.8 °C, and a frost-free period of 200 days. The entire plantation was subjected to unified furrow irrigation at the same time during July to August each year. All plants received the same fertilization regime. Base fertilizer was applied after fruit harvest, consisting of 2,500 kg/ha^-1^ decomposed farmyard manure plus 300 kg/ha^-1^ 15–15-15 sulfur-based compound fertilizer. Topdressing of 375 kg/ha^-1^ high-potassium compound fertilizer (12–18-15) was administered at the fruit expansion stage. Chemical control was performed uniformly across the orchard at the same time with the same pesticides and concentrations. All plants were managed under identical field practices.

### 2.2 Collection of soil sample

In September 2025, during the harvest period of *S. chinensis*, the experiment was carried out by dividing the field into three equal parts, representing the replicate plots S1, S2, and S3, with four parallel subplots (Sub1-Sub4) set in each region. A five-point sampling method was adopted to mark the sampling spots in each subplot (Supplementary Figure S2).

Healthy plants possessed intact and robust root systems with normal growth. Infected plants only showed reversible leaf wilting at noon. The root damage ratio of infected plants was 10–25%, mainly characterized by cortical browning, rot and light brown discoloration of vascular bundles. The main roots still retained partial physiological functions, indicating the early to middle stage of root rot. At this stage, roots were infected but still maintained partial functions, which could truly reflect early rhizosphere microbial changes caused by root rot and avoid interference from severe plant decline in the late stage. In addition, effective prevention and control could be conducted at the early to middle stage, while late-stage infection resulted in irreversible root necrosis. Therefore, this disease stage has sufficient rationality and practical value for the present study.

From each sampling spot, one naturally infected plant (at the early to middle stage of root rot) and one healthy plant were randomly selected, resulting in a total of five infected plants and five healthy plants per subplot. Then, from each selected plant, the soil attached loosely to the root surface was removed by shaking, and the rhizosphere soil adhering to the root surface (1–3 mm thick) was collected into sterile self-sealing bags under aseptic conditions. The rhizosphere soil samples collected from five plants of the same type in each subplot were mixed to form one replicate sample. Thus, four infected plant samples and four healthy plant samples were obtained from each region.

All samples were transported with ice packs to the laboratory within 2 h. The four samples from each region were pooled into one group, and finally, six target sample groups were obtained. The healthy groups (JK) were labeled as JK-1, JK-2, and JK-3, while the infected groups (BZ) were labeled as BZ-1, BZ-2, and BZ-3. Each sample was further divided into two subsamples: one was temporarily stored in an ultra-low temperature (−80 °C) freezer for metagenomic sequencing, and the other was air-dried for the determination of soil physicochemical properties.

### 2.3 Determination of soil physicochemical properties

The soil physicochemical properties were analyzed following the methods described by Lu (2000). Soil pH was measured using a soil-to-water suspension at a ratio of 1:2.5 (w:v) with the potentiometric method. Soil organic matter (OM) content was determined using the potassium dichromate oxidation method. Total nitrogen (TN) content was measured by the Kjeldahl method, and hydrolyzable nitrogen (HN) content was determined by colorimetry after extraction with KCl. Available phosphorus (AP) was extracted with 0.5 mol L^−1^ NaHCO₃ and determined by the molybdenum-antimony anti-colorimetric method. Total phosphorus (TP) was digested with HClO₄-H₂SO₄ and determined by the same colorimetric method. Available potassium (AK) was extracted with 1 mol L^−1^ NH₄OAc, while total potassium (TK) was digested by NaOH fusion. Both AK and TK were determined by flame photometry.

### 2.4 Determination of plant growth indicators

Plants used to collect the rhizosphere soil sample were adopted to analyze the growth indicators. The key growth indicators were measured before soil sampling. The natural extension length from the base of the main vine to the apical growing point was measured using a steel tape to obtain the main vine length. The diameter of the main vine was determined at a height of 20 cm above the ground in three different directions with a vernier caliper, and the average value was represented as the main vine diameter. Ten normally developed fruit spikes were randomly selected from each plant, and the length from the base to the apex of each spike was measured with a tape. The average value of the 10 spikes was represented as the fruit spike length. One hundred plump fresh berries were randomly selected from different fruit spikes of a single plant and weighed using an electronic balance to determine the 100-berry fresh weight. Further, at full maturity, all fresh fruits on a single plant were harvested and weighed after removing impurities to determine the fresh fruit yield per plant. A subsample of fresh fruits was oven-dried at 45 °C to a constant weight to determine the dried fruit yield per plant. The fruit drying ratio was calculated using the following Equation 1:


$$\begin{array}{l} \text{Drying ratio}=\text{Fresh fruit weight}\;\mathrm{per}\;\text{plant}/\text{Dried fruit}\;\\ \text{weight}\;\mathrm{per}\;\text{plant}\times 100\% \end{array}$$ (1)

### 2.5 Metagenomic sequencing and analysis

#### 2.5.1 DNA extraction, sequencing, and data quality control

Total genomic DNA of the rhizosphere microbial community was extracted using the E.Z.N.A.^®^ Soil DNA Kit (Omega Bio-tek, Inc., Norcross, GA, U.S.), following the manufacturer’s instructions. The concentration and purity of the extracted DNA were determined with TBS-380 and NanoDrop2000 (Thermo Fisher Scientific, Waltham, MA, USA), respectively, and the integrity was verified by 1% agarose gel electrophoresis. Further, DNA fragmentation was performed using a Covaris M220 instrument, and fragments of approximately 350 bp were selected for paired-end (PE) library construction. The libraries for sequencing were prepared with the NEXTFLEX Rapid DNA-Seq Kit (PerkinElmer, Inc., Waltham, MA, USA) using approximately 50 ng of DNA as input. PCR amplification during library preparation was performed with the following program: initial denaturation at 98 °C for 2 min, followed by 9 PCR cycles (denaturation at 98 °C for 30 s, annealing at 65 °C for 30 s, extension at 72 °C for 60 s), and a final extension at 72 °C for 4 min. Additionally, size selection of approximately 480 bp was conducted during library purification and metagenomic sequencing was conducted on the Illumina NovaSeq™ X Plus platform (Shanghai Majorbio Bio-pharm Technology Co., Ltd., Shanghai, China). The raw sequence data generated in this study have been deposited in the NCBI Sequence Read Archive under the accession number PRJNA1394726.

The adapter trimming and low-quality read filtering were both performed using fastp (version 0.20.0) (Chen et al., 2018; https://github.com/OpenGene/fastp). Adapter sequences at both the 5′ and 3′ ends of the reads were trimmed. Reads with a length shorter than 50 bp and an average base quality score below 20 were removed to generate high-quality clean reads for downstream analysis.

#### 2.5.2 Sequence assembly, gene prediction, and construction of a non-redundant gene set

High-quality clean reads were assembled using MEGAHIT (https://github.com/voutcn/megahit, version 1.1.2) (Li et al., 2015), and contigs longer than 300 bp were selected to obtain the final assembly. The open reading frames (ORFs) in the assembled contigs were predicted using Prodigal (https://github.com/hyattpd/Prodigal, version 2.6.3) (Hyatt et al., 2010). Finally, genes longer than 100 bp were selected and translated into amino acid sequences using Emboss 6.6.0 (http://emboss.open-bio.org/, V6.6.0). All gene sequences predicted for the samples were clustered with CD-HIT (http://weizhongli-lab.org/cd-hit/, version 4.7) (Fu et al., 2012), using 90% identity and 90% coverage to construct a non-redundant gene catalog.

#### 2.5.3 Gene abundance calculation and taxonomic annotation

High-quality clean reads obtained from each sample were mapped to the non-redundant gene catalog using SOAPaligner (https://github.com/ShujiaHuang/SOAPaligner, soap2.21 version) with a sequence identity threshold set as 95% (Li et al., 2008). Further, the amino acid sequences of the non-redundant gene catalog were aligned against the NR database (Non-Redundant Protein) using DIAMOND (https://github.com/bbuchfink/diamond, version 2.0.13), with the e-value cutoff set as 1e^−5^ (Buchfink et al., 2015). Taxonomic annotation was obtained from the taxonomic database corresponding to the NR database. Finally, the abundance of each taxon was calculated as the sum of the abundances of all genes annotated to that taxon.

#### 2.5.4 Functional annotation

The amino acid sequences of the non-redundant gene catalog were aligned against the eggNOG database using DIAMOND (https://github.com/bbuchfink/diamond, version 2.0.13), with the e-value cutoff set as 1e^−5^ (Buchfink et al., 2015), to obtain the Clusters of Orthologous Groups (COG) functional annotations of the genes. Then, the abundance of each COG category was calculated as the sum of the abundances of all genes annotated to that category.

The amino acid sequences of the non-redundant gene catalog were aligned against the Kyoto Encyclopedia of Genes and Genomes (KEGG) database using DIAMOND (https://github.com/bbuchfink/diamond, version 2.0.13), with the e-value cutoff set as 1e^−5^ (Buchfink et al., 2015), to obtain the KEGG functional annotations of the genes. The abundance of each KEGG pathway was calculated as the sum of the abundances of all genes annotated to that pathway.

The amino acid sequences of the non-redundant gene catalog were aligned against the CAZy database using hmmscan,^1^ with the e-value cutoff catalog as 1e^−5^, to obtain the annotation information of the carbohydrate-active enzymes.^2^ The abundance of each CAZy category was calculated as the sum of the abundances of all genes annotated to that category.

### 2.6 Statistical analysis

Alpha diversity indices of the microbial community in the rhizosphere were calculated using mothur (version v.1.30.2, https://mothur.org/wiki/calculators/). Non-metric multidimensional scaling (NMDS), analysis of similarities (ANOSIM), variance inflation factor (VIF) analysis, and redundancy analysis (RDA) were performed using the vegan package (version 2.4.3) in R software (version 3.3.1). NMDS was performed to examine whether there were differences in bacterial community composition between treatments, with the arrangement of sample points representing the similarity among individual samples. ANOSIM was conducted to test whether the differences between treatments were greater than the differences among replicates. Linear discriminant analysis (LDA) effect size (LEfSe, http://galaxy.biobakery.org/) was applied to identify the differentially abundant microbial taxa between groups based on taxonomic composition and uncover potential microbial indicators linked to soil health and root rot occurrence.

All data were expressed as mean ± standard deviation (SD; *n* = 3). Statistical analyses were performed using SPSS software (version 27.0). Differences between the two groups were assessed using an independent samples *t*-test (*p* < 0.05). The graphs were plotted using Origin 2022 software.

## 3 Results

### 3.1 Effects of root rot on the growth of *S. chinensis*

The incidence of root rot significantly inhibited the growth and yield of *S. chinensis* (Table 1). The main vine length and diameter of infected *S. chinensis* plants demonstrated 35.81 and 23.72% decrease, respectively, compared with the healthy plants. The fruit-bearing traits and yield indicators of the infected group were also significantly inferior to those of the healthy group. The fruit spike length, 100-berry fresh weight, fresh fruit yield per plant, dried fruit yield per plant, and drying ratio of the infected group decreased by 48.40, 33.23, 70.51, 77.78, and 24.64%, respectively, compared with the healthy group.

**Table 1:** Growth and yield traits of healthy and infected *Schisandra chinensis*.

Group	Main vine length (m)	Main vine diameter (cm)	Fruit spike length (cm)	100-berry fresh weight (g)	Fresh fruit yield per plant (kg)	Dried fruit yield per plant (kg)	Drying ratio (%)
JK	3.63 ± 0.21^a^	1.56 ± 0.^09a^	10.60 ± 0.75^a^	47.73 ± 1.68^a^	3.56 ± 0.07^a^	0.90 ± 0.04^a^	25.37 ± 0.74^a^
BZ	2.33 ± 0.25^b^	1.19 ± 0.15^b^	5.47 ± 0.67 ^b^	31.87 ± 1.66^b^	1.06 ± 0.23^b^	0.20 ± 0.04^b^	19.12 ± 0.13^b^

Data represent means ± SD (n = 3). Different lowercase letters in the same column indicate significant differences (p < 0.05). JK—healthy group; BZ—infected group.

### 3.2 Physicochemical properties of rhizosphere soil

**Figure 1:**
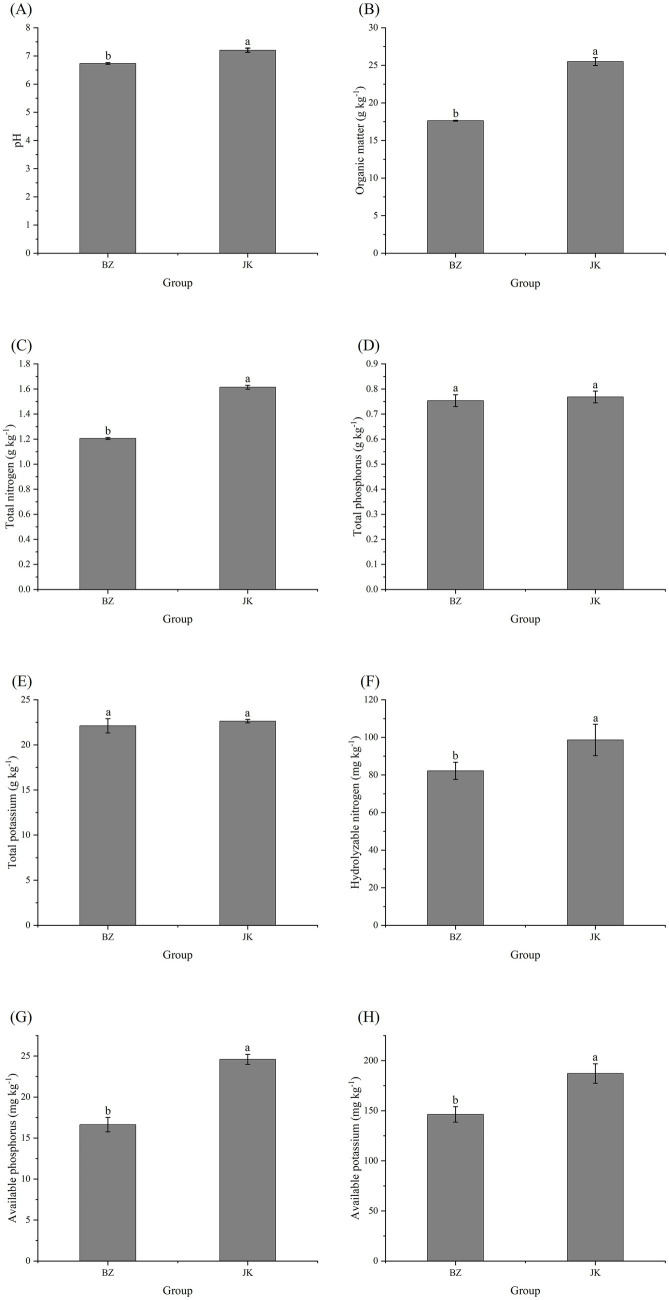
Physicochemical properties of rhizosphere soils from healthy and infected *Schisandra chinensis*. **(A)** Soil pH; **(B)** organic matter content; **(C)** total nitrogen content; **(D)** total phosphorus content; **(E)** total potassium content; **(F)** Hydrolyzable nitrogen content; **(G)** available phosphorus content; **(H)** available potassium content. Different lowercase letters above the bars indicate significant differences between treatments (*p* < 0.05). JK—healthy group; BZ—infected group.

Further analysis revealed that the rhizosphere soil of infected *S. chinensis* plants was acidic, while that of healthy plants was alkaline. The contents of soil organic matter, total nitrogen, hydrolyzable nitrogen, available phosphorus, and available potassium in the rhizosphere soil of infected *S. chinensis* plants were significantly lower than those of the healthy plants (*p* < 0.05; Figure 1). These observations indicated that the occurrence of root rot could induce rhizosphere soil acidification and significantly reduce soil organic matter and the available contents of nitrogen, phosphorus, and potassium.

### 3.3 Rarefaction curves based on the Shannon index

To assess whether the sequencing depth was sufficient to capture the bacterial diversity in the rhizosphere soil of *S. chinensis*, rarefaction curves based on the Shannon index were generated for all samples (Supplementary Figure S3). As shown in the figure, the Shannon index curves for all samples rose rapidly at the initial stage with the increase of sequencing reads, and then gradually plateaued when the number of sequences exceeded approximately 500,000. This clear asymptotic trend indicates that the sequencing depth was adequate to cover the majority of bacterial taxa in each sample, and further increasing the number of reads would not significantly improve the observed microbial diversity. Therefore, the differences in bacterial community diversity between the healthy and diseased groups were not driven by uneven or insufficient sequencing depth, confirming the reliability of subsequent diversity analyses.

### 3.4 Bacterial diversity of rhizosphere soil

#### 3.4.1 Alpha diversity

**Figure 2:**
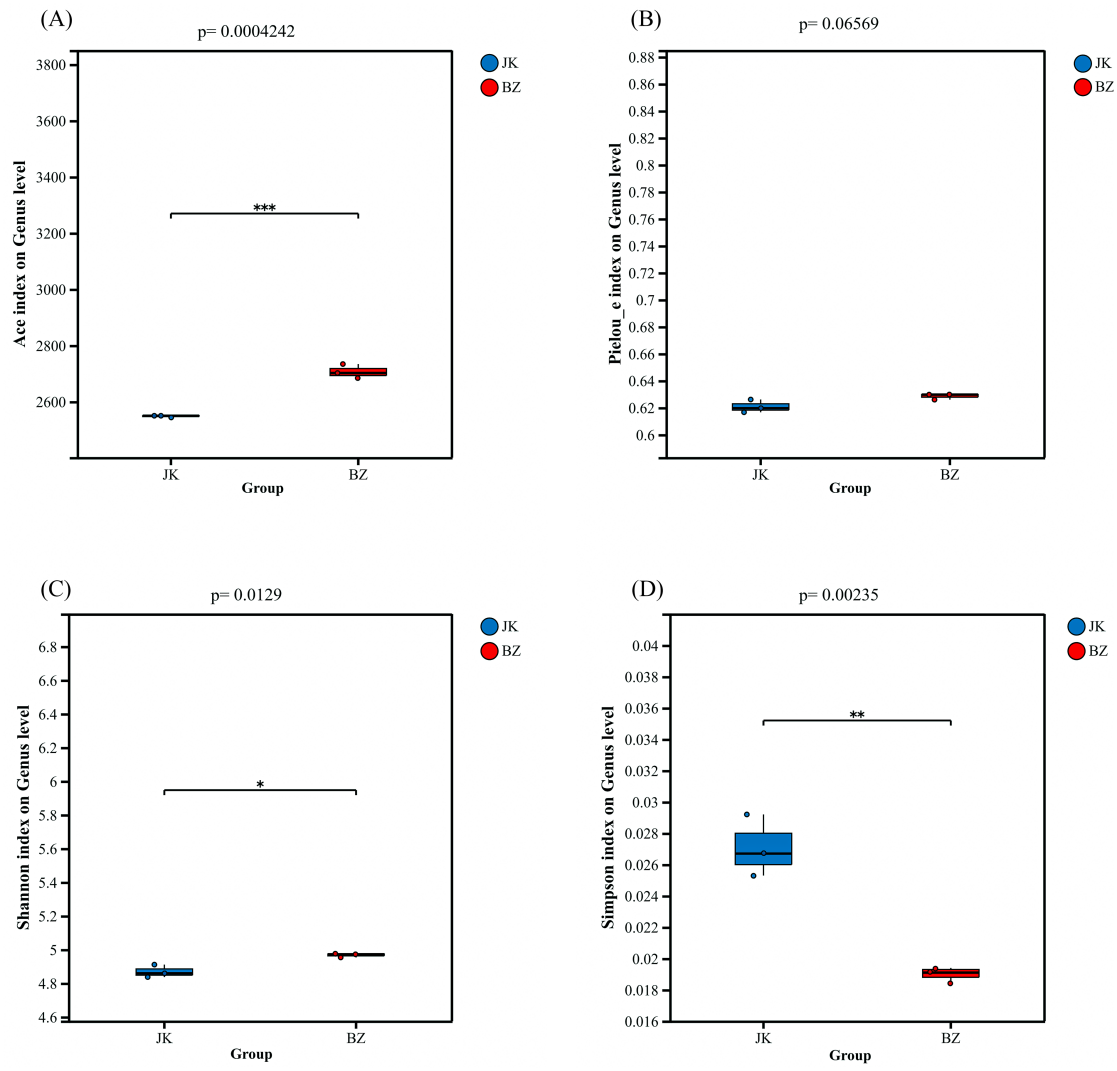
Alpha diversity of rhizosphere soil microbial communities between healthy and infected *Schisandra chinensis*. **(A)** Ace index; **(B)** Pielou_e index; **(C)** Shannon index; **(D)** Simpson index. JK—healthy group; BZ—infected group.

The Ace index of the bacterial community in the rhizosphere soil of infected *S. chinensis* plants was significantly higher than that of the healthy plants (*p* < 0.05; Figure 2). These observations indicated greater bacterial species richness in the rhizosphere soil of infected *S. chinensis* than in the healthy group. Similarly, the Shannon index of the bacterial community in the rhizosphere soil of the infected group was higher than that of the healthy group (*p* < 0.05; Figure 2). In contrast, the Simpson index of the healthy group was higher than that of the infected group (*p* < 0.05; Figure 2). These results suggested greater diversity of bacterial species in the rhizosphere soil of infected *S. chinensis* than in the healthy group.

#### 3.4.2 Beta diversity

**Figure 3:**
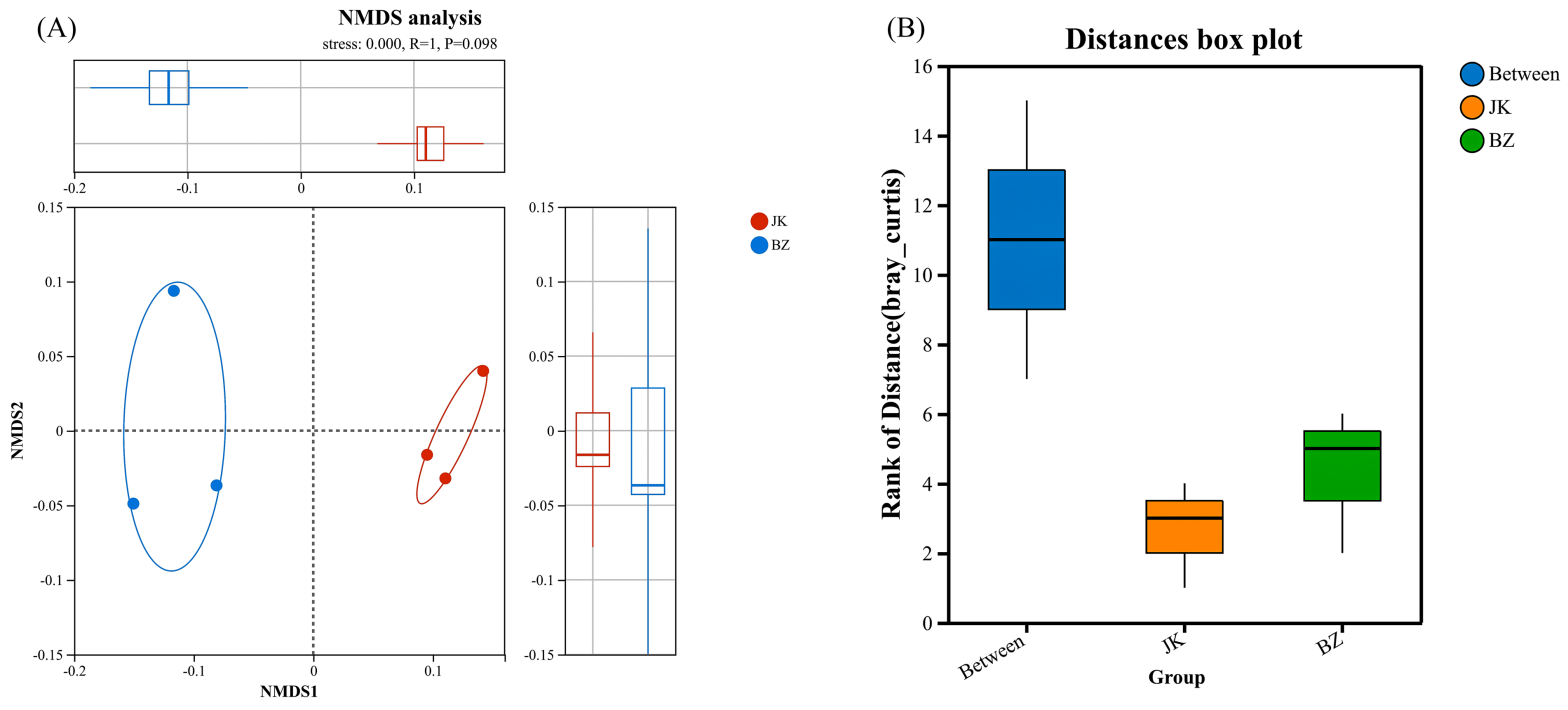
Non-metric multidimensional scaling (NMDS) and ANOSIM analysis of rhizosphere bacterial communities from healthy and infected *Schisandra chinensis*. **(A)** NMDS ordination plot of rhizosphere bacterial communities; **(B)** ANOSIM analysis boxplot showing the rank of Bray-Curtis distances between and within groups. JK—healthy group; BZ—infected group.

The NMDS revealed separation trends in the rhizosphere soil bacterial community structure between healthy and root rot-infected *S. chinensis* plants (Figure 3). The ANOSIM further indicated a clear divergence trend in community structure between the JK and BZ groups (Figure 3). Overall, the rhizosphere microhabitats of healthy and infected *S. chinensis* plants exhibited a tendency of differentiation.

### 3.5 Variations in the composition and abundance of rhizosphere soil bacterial communities between healthy and root rot-infected *Schisandra chinensis*

**Figure 4:**
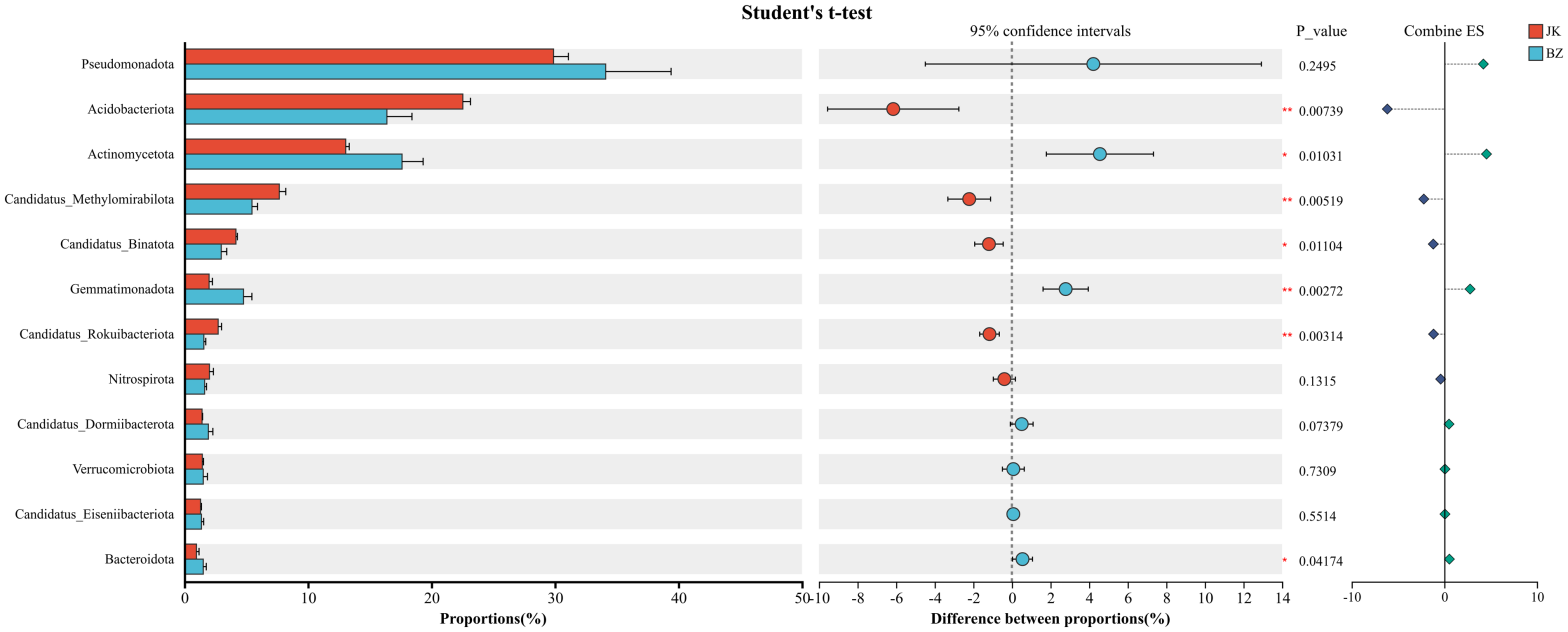
Differences in rhizosphere bacterial phylum level composition between healthy and infected *Schisandra chinensis*. JK—healthy group; BZ—infected group.

A total of 154 bacterial phyla were detected in the rhizosphere soil of healthy *S. chinensis* plants, while 162 were identified in the rhizosphere soil of infected plants. The top 12 bacterial phyla in terms of relative abundance in both groups (Supplementary Figure S4) were Pseudomonadota (29.86, 34.13%), Acidobacteriota (22.54, 16.38%), Actinomycetota (13.06, 17.62%), Candidatus_Methylomirabilota (7.69, 5.46%), Candidatus_Binatota (4.17, 2.98%), Gemmatimonadota (1.99, 4.78%), Candidatus_Rokubacteriota (2.74, 1.57%), Nitrospirota (2.02, 1.63%), Candidatus_Dormibacterota (1.42, 1.93%), Verrucomicrobiota (1.44, 1.52%), Candidatus_Eiseniibacteriota (1.3, 1.52%), and Bacteroidota (0.96, 1.52%). The dominant bacterial phyla in the rhizosphere soil of the healthy group and the infected group were the same, namely Pseudomonadota, Acidobacteriota, and Actinomycetota, but the relative abundance of each phylum varied between the two groups (Figure 4). The infected group showed higher relative abundances of Actinomycetota, Gemmatimonadota, and Bacteroidota than the healthy group. In contrast, the healthy group had higher relative abundances of Acidobacteriota, Candidatus_Methylomirabilota, Candidatus_Binatota, and Candidatus_Rokubacteriota than the infected group.

**Figure 5:**
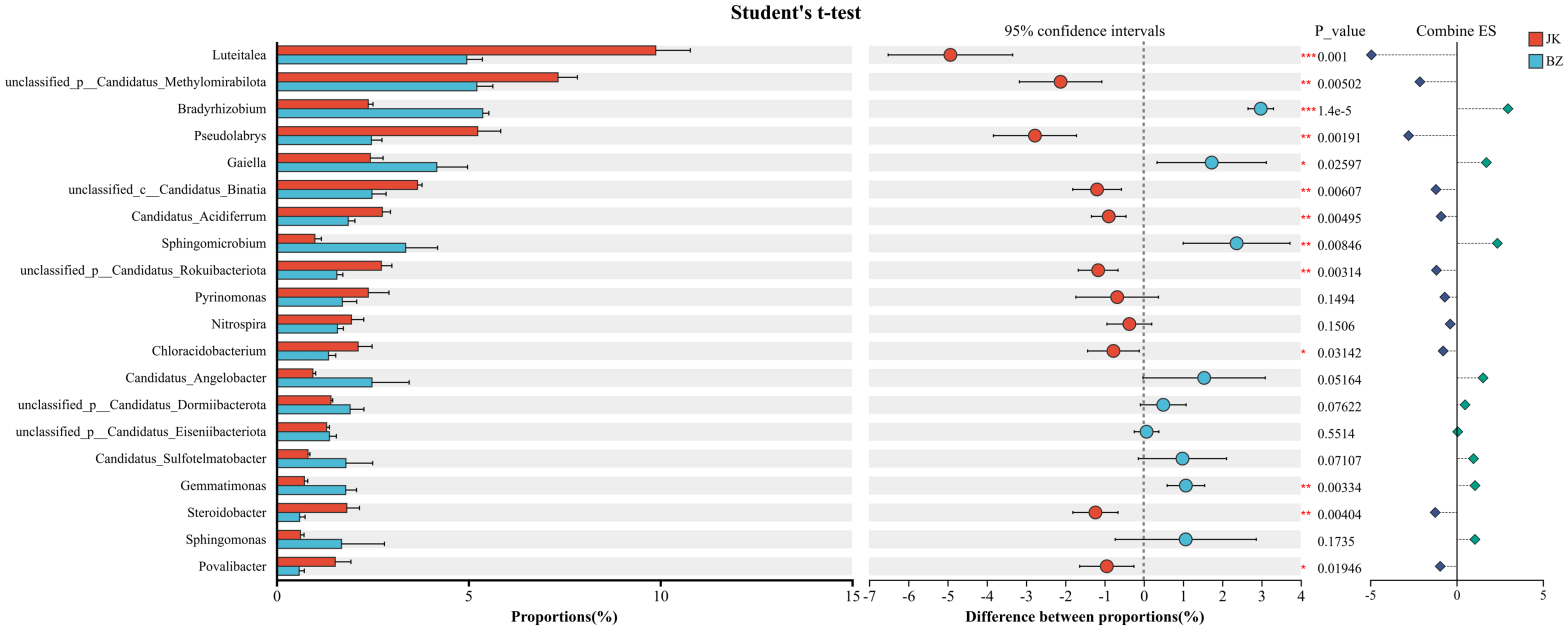
Differences in rhizosphere bacterial genus level composition between healthy and infected *Schisandra chinensis*. JK—healthy group; BZ—infected group.

At the genus level, the top 20 bacterial genera in relative abundance accounted for only 36.72 and 35.90% of the entire bacterial community in the healthy group and the infected group, respectively (Supplementary Figure S5). These genera were *Luteitalea* (9.88, 4.95%), Candidatus_Methylomirabilota (7.33, 5.22%), *Bradyrhizobium* (2.39, 5.37%), *Pseudolabrys* (5.24, 2.47%), *Gaiella* (2.44, 4.17%), Candidatus_Binatia (3.67, 2.48%), Candidatus_Acidiferrum (2.75, 1.86%), *Sphingomicrobium* (0.99, 3.36%), Candidatus_Rokuibacteriota (2.73, 1.57%), *Pyrinomonas* (2.39, 1.71%), *Nitrospira* (1.95, 1.58%), *Chloracidobacterium* (2.12, 1.35%), Candidatus_Angelobacter (0.94, 2.48%), Candidatus_Dormiibacterota (1.41, 1.91%), Candidatus_Eiseniibacteriota (1.30, 1.37%), Candidatus_Sulfotelmatobacter (0.82, 1.80%), *Gemmatimonas* (0.72, 1.80%), *Steroidobacter* (1.83, 0.60%), *Sphingomonas* (0.62, 1.69%), and *Povalibacter* (1.53, 0.59%). Consistent with the phylum-level pattern, the core dominant genera were shared between the two groups, but their relative abundances varied markedly (Figure 5). The infected group had significantly higher relative abundances of *Bradyrhizobium*, *Gaiella*, *Sphingomicrobium*, and *Gemmatimonas* than the healthy group. In contrast, the healthy group was significantly enriched in *Luteitalea*, Candidatus_Methylomirabilota, *Pseudolabrys*, Candidatus_Binatota, Candidatus_Acidiferrum, Candidatus_Rokuibacteriota, *Chloracidobacterium*, *Steroidobacter*, and *Povalibacter*.

### 3.6 LEfSe screening of rhizosphere bacterial biomarkers in healthy and root rot-infected *S. chinensis*

**Figure 6:**
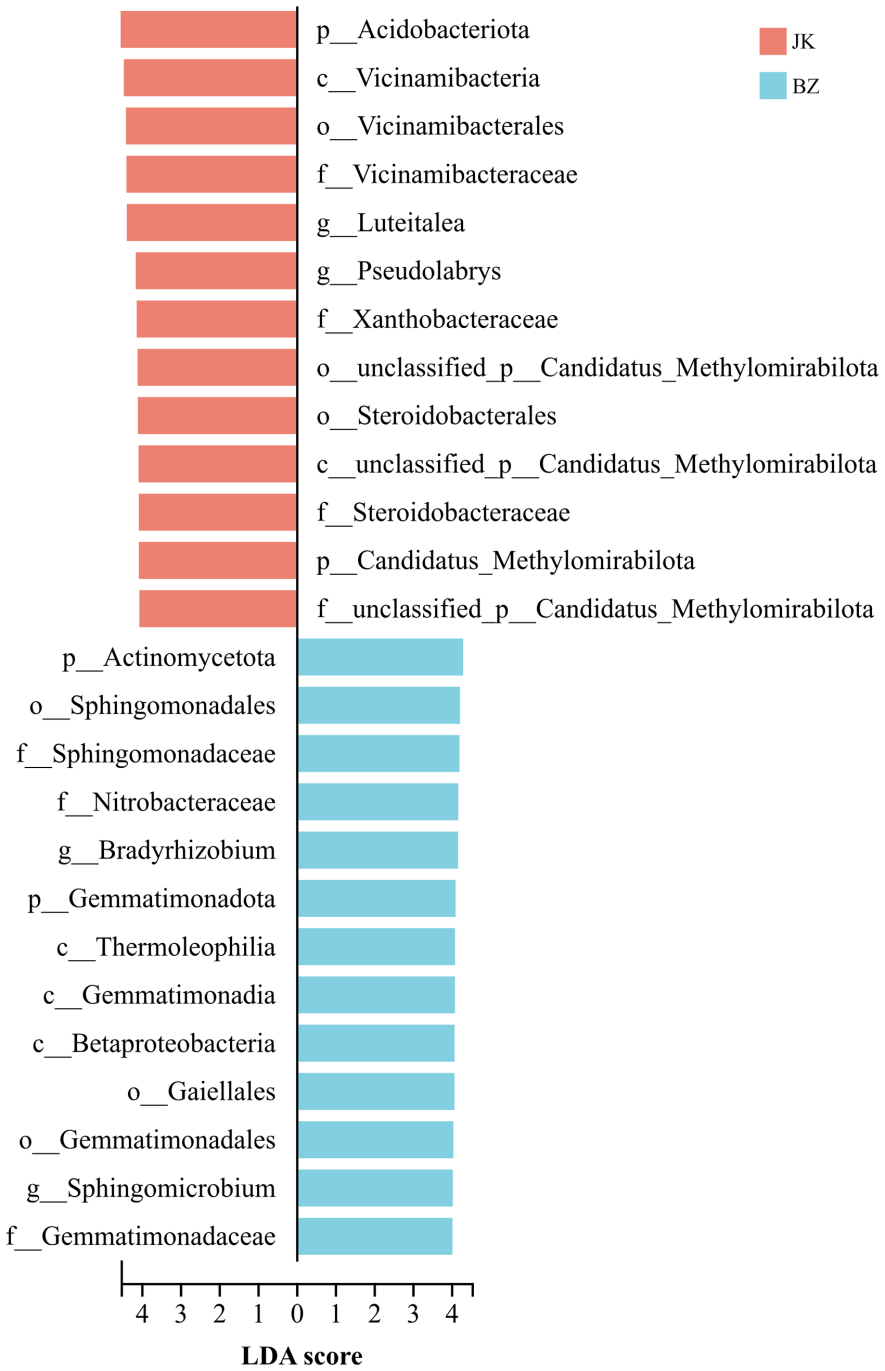
LEfSe analysis of rhizosphere bacterial communities from healthy and infected *Schisandra chinensis* (from phylum to genus level). JK—healthy group; BZ—infected group.

Subsequently, LEfSe analysis (LDA > 4, *p* < 0.05) was used to screen the microorganisms from the phylum to genus levels with significant differences between the two groups (Figure 6). A total of 26 enriched bacterial taxa were identified, with 13 taxa enriched in the rhizosphere soil of the healthy group and 13 in that of the infected group. The characteristic differential taxa specific to the healthy group were predominantly concentrated in the phylum Acidobacteriota, encompassing taxa at the class (Vicinamibacteria), order (Vicinamibacterales), family (Vicinamibacteraceae), and genus (*Luteitalea*) levels. Additionally, *Pseudolabrys* belonging to Xanthobacteraceae in Pseudomonadota, as well as some unclassified taxa under Candidatus_Methylomirabilota, were significantly enriched in the healthy group. In contrast, Actinomycetota, Pseudomonadota, and Gemmatimonadota were enriched in the infected group. The bacteria of these groups mainly included *Bradyrhizobium* and *Sphingomicrobium* belonging to Alphaproteobacteria in Pseudomonadota, Gemmatimonadaceae in Gemmatimonadota, and Gaiellales in Actinomycetota. In summary, the study detected significant differences in the enriched taxa between the healthy group and the infected group.

### 3.7 Functional annotations of rhizosphere soil bacterial communities

#### 3.7.1 COG functional annotation of rhizosphere bacteria and intergroup analysis of differentially expressed functional genes

**Figure 7:**
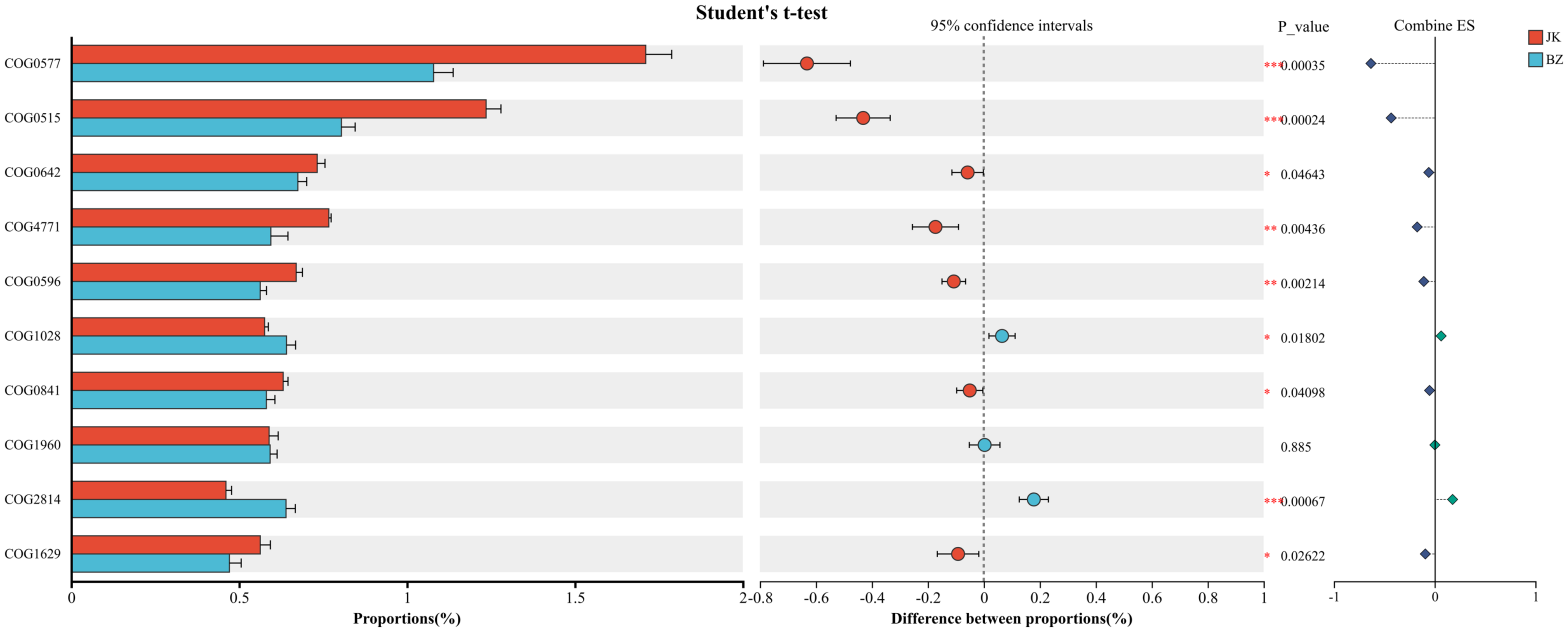
Intergroup comparative analysis of differentially enriched COG functional genes in rhizosphere bacteria between healthy and infected *Schisandra chinensis*. JK—healthy group; BZ—infected group.

COG functional annotation revealed that amino acid transport and metabolism, energy production and conversion, signal transduction mechanisms, and carbohydrate transport and metabolism were the main functions associated with the *S. chinensis* rhizosphere bacteria (Supplementary Figure S6). Furthermore, Student’s t-test was performed to analyze the intergroup differences in the abundance of the top 10 core COG functional genes and confirm the reliability (Figure 7). The abundances of seven genes, including COG0577, COG0515, COG4771, and COG0596, were significantly high in the healthy group (*p* < 0.05), while those of COG2814 and COG1028 were significantly high in the infected group (*p* < 0.05).

Finally, based on LEfSe analysis, the core functional markers with inter-group discrimination ability were screened from the differential genes (Supplementary Figure S7). The functional gene clusters significantly enriched by the rhizosphere soil bacteria of healthy *S. chinensis* included COG4993, COG4771, COG0596, and COG1529, whereas that significantly enriched in the rhizosphere soil bacteria of infected *S. chinensis* was COG2814. In summary, the core function of bacteria in the *S. chinensis* rhizosphere was amino acid transport and metabolism. However, significant differences were detected in the functional gene abundance patterns of bacteria between the healthy group and the infected group, while significantly less diverse metabolic pathways were detected for the infected group, which had fewer enriched pathways than the healthy group.

#### 3.7.2 KEGG and CAZy functional annotation of rhizosphere bacteria and intergroup analysis

**Figure 8:**
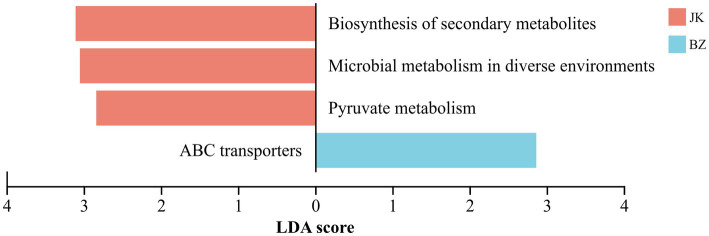
LEfSe analysis of KEGG metabolic pathways in rhizosphere bacterial communities between healthy and infected *Schisandra chinensis*. JK—healthy group; BZ—infected group.

KEGG enrichment analysis on the bacterial community of *S. chinensis* rhizosphere soil (LDA > 2.8) identified four differential metabolic pathways (Figure 8). Among them, three pathways were identified in the rhizosphere soil of healthy *S. chinensis* plants, and one in that of the infected plants. The key differential metabolic pathways of the healthy group were biosynthesis of secondary metabolites, microbial metabolism in diverse environments, and pyruvate metabolism, while that of the rhizosphere soil microorganisms of infected *S. chinensis* was ABC transporters. These results indicated that the occurrence of root rot significantly altered the key metabolic functions of the *S. chinensis* rhizosphere bacterial community.

**Figure 9:**
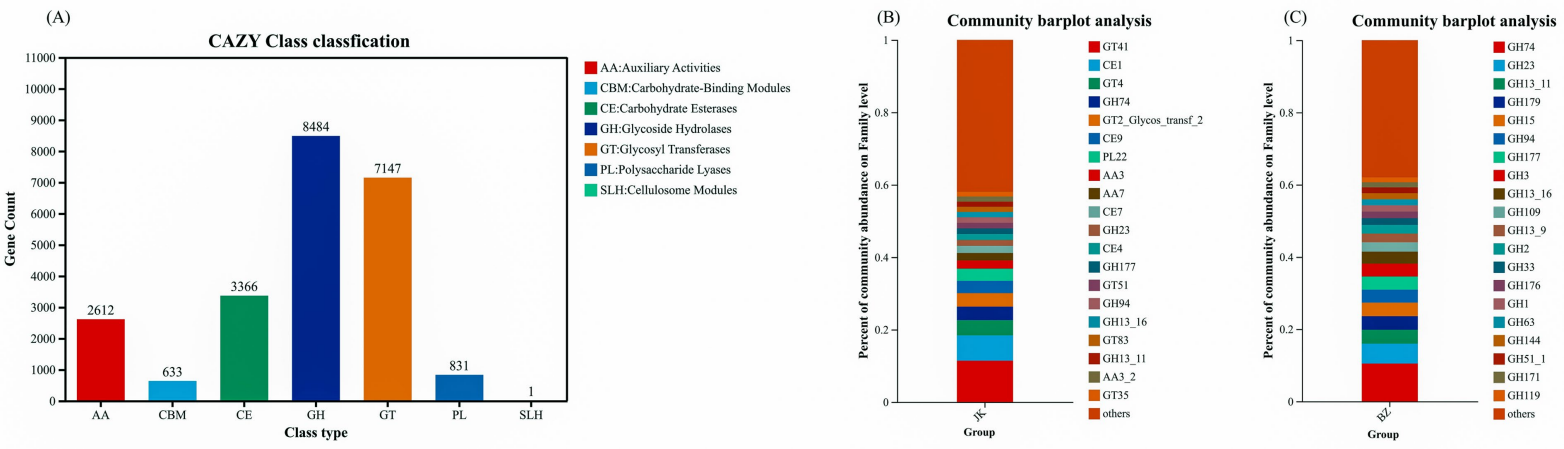
CAZy annotation and inter-group differential analysis of carbohydrate-active enzymes. **(A)** CAZy classfication; **(B)** community barplot analysis of healthy group; **(C)** community barplot analysis of infected group. JK—healthy group; BZ—infected group.

Furthermore, CAZy functional annotation revealed that among the carbohydrate metabolism-related genes enriched by the *S. chinensis* rhizosphere soil bacteria (Figure 9), glycoside hydrolases (GH) accounted for the largest proportion (36.76%), followed by glycosyl transferases (GT) (30.97%). The top 20 enzymes in terms of abundance in the healthy group included GT, GH, carbohydrate esterases (CE), polysaccharide lyases (PL), and auxiliary activities (AA), among which the top three were GT41, CE4, and GT4 (Figure 9). Meanwhile, all the top 20 enzymes enriched by the infected group were of the GH category (Figure 9). These observations indicated that the occurrence of root rot significantly altered the composition and distribution characteristics of enzymes related to carbohydrate metabolism in the rhizosphere bacteria of *S. chinensis*.

### 3.8 Relationships between rhizosphere bacterial communities and environmental factors

To further identify the environmental factors driving the changes in the rhizosphere soil microbial communities of healthy and infected *S. chinensis* plants, redundancy analysis was conducted for the dominant bacterial communities at the phylum level and the environmental factors. The major environmental factors were screened based on the VIF (VIF > 10) (Supplementary Table S1). The approach indicated that total phosphorus, total potassium, hydrolyzable nitrogen, and available potassium were significantly correlated with the rhizosphere soil bacterial community structure.

**Figure 10:**
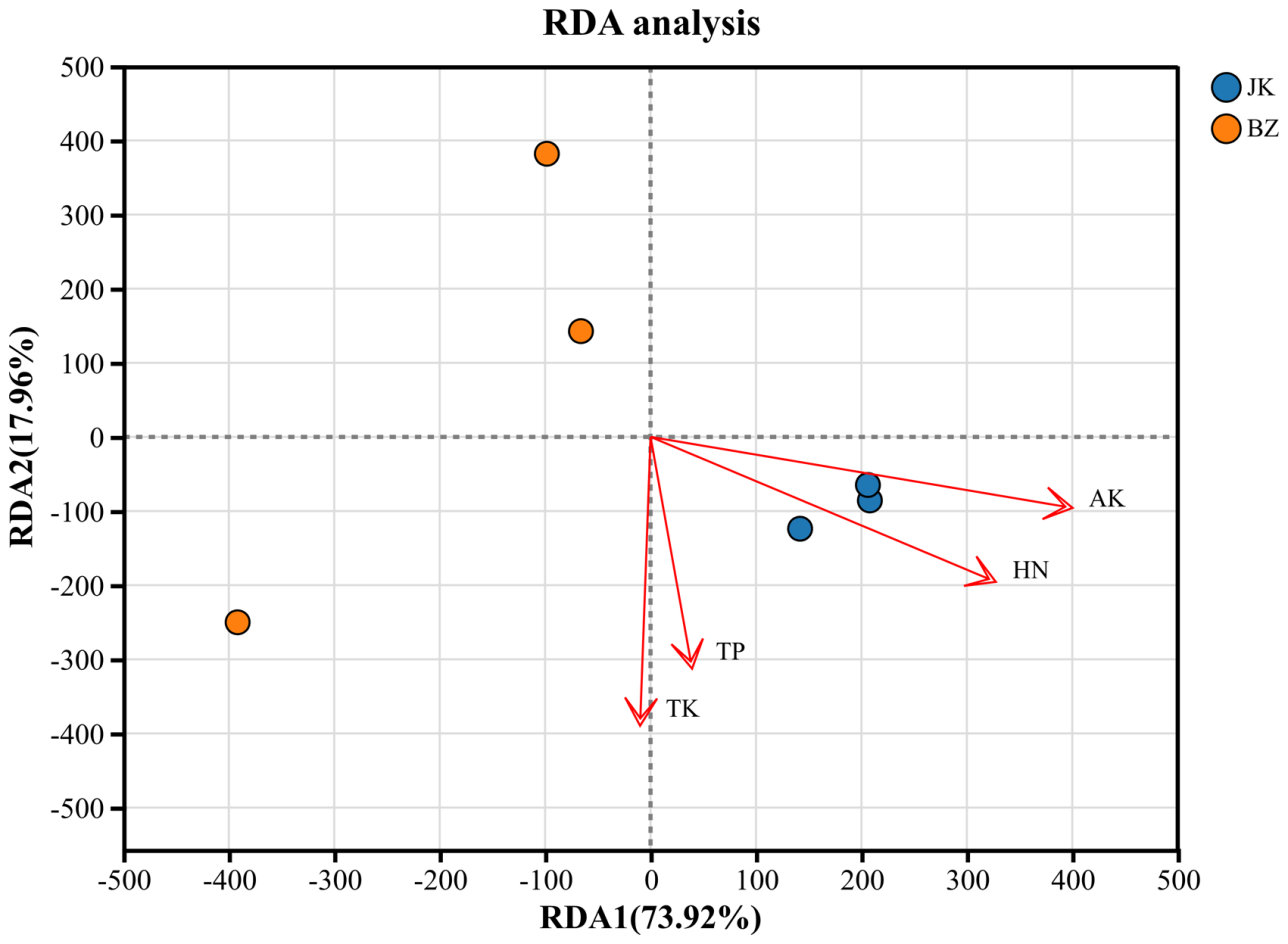
Redundancy analysis of rhizosphere soil bacterial taxa and environmental factors. JK—healthy group; BZ—infected group.

The first and second ordination axes explained 73.92 and 17.96% of the variations in the bacterial diversity (Figure 10), and the selected soil environmental factors together explained 91.88% of the total eigenvalues. Hydrolyzable nitrogen and available potassium exhibited a strong positive correlation with the bacterial community structure of the healthy group (the arrows pointed to the sample area of the healthy group). In contrast, total phosphorus and total potassium were more closely related to the community structure of the infected group (the arrows were closer to the sample area of the infected group). Combined with the results of soil physicochemical property analysis, we found that the contents of HN and AK in the healthy group were significantly higher than those in the infected group, which further confirmed that hydrolyzable nitrogen and available potassium were the core environmental factors driving the differentiation in rhizosphere bacterial community structure between healthy and infected *S. chinensis* plants.

Pearson correlation analysis was further conducted to explore the correlations among environmental factors, bacterial communities, diversity indices and functional characteristics. We found that hydrolyzable nitrogen was positively correlated with the Simpson index, the abundances of Acidobacteriota, Gaiellales, *Pseudolabrys*, COG4993, COG4771, COG0596, and COG1529, and biosynthesis of secondary metabolites (Supplementary Figure S8). On the contrary, it was negatively correlated with Ace index, Shannon index, *Actinomycetota*, *Bradyrhizobium*, and COG2814. Available potassium was positively correlated with the Simpson index and the abundances of Acidobacteriota, Candidatus_Methylomirabilota, Xanthobacteraceae, *Pseudolabrys*, COG4993, COG4771, COG0596, COG1529, biosynthesis of secondary metabolites, microbial metabolism in diverse environments, and pyruvate metabolism abundance. Meanwhile, it was negatively correlated with Ace index, Shannon index, Gemmatimonadota, Bradyrhizobium, Sphingomicrobium, and COG2814 abundance.

Ace index was positively correlated with the abundances of Actinomycetota, Gemmatimonadota, Gaiellales, *Bradyrhizobium*, *Sphingomicrobium*, COG2814, and ABC transporters. In contrast, they were negatively correlated with the abundances of Acidobacteriota, Candidatus_Methylomirabilota, Xanthobacteraceae, *Pseudolabrys*, COG4993, COG4771, COG0596, COG1529, biosynthesis of secondary metabolites, microbial metabolism in diverse environments, and pyruvate metabolism. The Shannon index was positively correlated with the abundances of Gemmatimonadota, *Bradyrhizobium*, *Sphingomicrobium*, and COG2814, while it was negatively correlated with the abundances of Acidobacteriota, Candidatus_Methylomirabilota, Xanthobacteraceae, *Pseudolabrys*, COG4993, COG0596, COG1529, biosynthesis of secondary metabolites, microbial metabolism in diverse environments, and pyruvate metabolism. Simpson index was positively correlated with the abundances of Acidobacteriota, Candidatus_Methylomirabilota, Xanthobacteraceae, *Pseudolabrys*, COG4993, COG4771, COG0596, COG1529, biosynthesis of secondary metabolites, microbial metabolism in diverse environments, and pyruvate metabolism. Meanwhile, it was negatively correlated with the abundances of Actinomycetota, Gemmatimonadota, *Bradyrhizobium*, *Sphingomicrobium*, and COG2814.

## 4 Discussion

### 4.1 Response of rhizosphere soil nutrient features to root rot infection in *Schisandra chinensis*

The occurrence of root rot significantly inhibited *S. chinensis* growth and yield. Typically, soil physicochemical properties serve as the core foundation for maintaining healthy plant growth and rhizosphere microbial community balance (Xie et al., 2023). The nutrient composition of the soil dynamically changes with plant health status, thereby affecting the structural composition and species richness of soil microbial communities. The present study correlated hydrolyzable nitrogen (HN) and available potassium (AK) positively with the Simpson index but negatively with the Ace index and Shannon index. This observation indicates that hydrolyzable nitrogen and available potassium are important soil nutrient factors regulating the diversity and species richness of the rhizosphere bacterial community in *S. chinensis*, and changes in their contents directly affect the structural characteristics of the rhizosphere bacterial community.

Research has established a close correlation between plant disease occurrence and soil physicochemical properties (Jamil et al., 2022; Martín-Cardoso and San Segundo, 2025). For instance, potassium in soil enhances plant environmental adaptability and stress resistance, and appropriate application of potassium fertilizer improves plant resistance to pathogenic fungi such as *Fusarium* spp. (Brandano et al., 2023; Cai et al., 2020). This study found that the rhizosphere soil of healthy plants was weakly alkaline, whereas that of infected plants was acidic. Additionally, the contents of organic matter, total nitrogen, hydrolyzable nitrogen, available phosphorus, and available potassium in the rhizosphere soil of the infected plants were significantly lower than those in the healthy plants. Generally, soil acidification and nutrient deficiencies exacerbate disease occurrence (Muneer et al., 2022). The acidic environment reduced soil nutrient availability (Jing et al., 2024), leading to an insufficient supply of nitrogen, phosphorus, potassium, and other nutrients for *S. chinensis*, and ultimately decreasing stress resistance. Nutrient deficiency disrupted the stability of the rhizosphere microenvironment (Wang et al., 2025), inhibited the multiplication of beneficial microorganisms, and created favorable conditions for pathogen infection. RDA confirmed HN and AK as the core environmental factors shaping the rhizosphere bacterial community structure of *S. chinensis*, consistent with previous research findings (Wang et al., 2025); these findings thus underscore that soil HN and AK are key edaphic drivers of rhizosphere bacterial community assembly and validate a tight regulatory relationship between soil nutrients and rhizosphere microbiota in *S. chinensis*.

### 4.2 Differential response of bacterial community structure and core taxa of the *Schisandra chinensis* rhizosphere to root rot infection

After pathogens infect plants, the rhizosphere microbial community structure changes (Jiang et al., 2019). Studies on Chinese cabbage soft rot have shown that infection by the pathogen *Brassica rapa* subsp*. pekinensis* leads to significant differences in the composition of rhizosphere bacterial and fungal communities between healthy and diseased plants (Li et al., 2024). Similarly, previous studies on strawberry root rot have demonstrated that root rot infection altered microbial diversity, changed rhizosphere microbial community composition, reduced the stability of microbial interaction networks, and enriched pathogenic and saprophytic microbes (Zhang M. et al., 2023). Studies on American ginseng root rot have also reported that root rot occurrence leads to significant changes in soil physicochemical properties and microbial community composition (Tian et al., 2021). In this study, the differences in rhizosphere soil properties and microbial community structure are closely related to root rot infection. Through systematic analysis, it is confirmed that the occurrence of root rot is the primary factor leading to changes in soil physicochemical properties and microbial community composition. Changes in rhizosphere microbial composition serve as the early warning signs of bacterial wilt of tomato (Gu et al., 2022), and the occurrence of *Panax notoginseng* disease is associated with decreased soil microbial diversity (Wu et al., 2015). Similarly, in this study, alpha diversity analysis showed that the richness and diversity of soil bacteria in the rhizosphere of infected *S. chinensis* were significantly higher than those in the healthy group, which contrasts with the conclusion of Zhou et al. (2019), who investigated microbial diversity in the rhizosphere soil of Fusarium wilt-affected banana and disease-free banana plants. However, subsequent analysis revealed that hydrolyzable nitrogen and available potassium were negatively correlated with diversity indices such as ACE, indicating that the deficiency of key nutrients (N and K) caused by root rot disrupted the stability of the bacterial community and created colonization niches for low-nutrient-tolerant opportunistic microorganisms, which further multiplied and supplemented into the community, thereby increasing species richness (Liu et al., 2024).

Further correlation analysis showed that the Ace index was positively correlated with the abundances of Actinomycetota, Gemmatimonadota, Gaiellales, *Bradyrhizobium*, and *Sphingomicrobium*, indicating that the infected group increased species richness by enriching these microbial groups. Additionally, NMDS and ANOSIM proved a tendency of differentiation in rhizosphere bacterial community structure between healthy and infected *S. chinensis*, suggesting that root rot occurrence triggers a fundamental transformation of the rhizosphere microbial flora (Liu et al., 2024; Zhou et al., 2019).

Rhizosphere bacterial community shows certain similarities in composition across different environments and soil types (Malviya et al., 2022). As common dominant groups in soil microorganisms, Pseudomonadota and Acidobacteriota usually have similar abundance levels across a wide range of soil environments (Chen et al., 2019). The present study found that the dominant bacterial phyla in the rhizosphere soil of healthy and infected *S. chinensis* were basically the same, with Pseudomonadota, Acidobacteriota, and Actinomycetota being the common and predominant phyla in both groups. These conserved and shared taxa may potentially constitute the stable core microbiome of the *S. chinensis* rhizosphere, which maintains fundamental ecological functions regardless of plant health status. These findings are consistent with existing research conclusions on the effects of tree species on soil microbial communities (Chen et al., 2019). Pseudomonadota, as typical copiotrophic bacteria, thrive in nutrient-rich rhizosphere environments and play a central role in soil biogeochemical cycling, thereby supporting plant nutrient acquisition and maintaining soil fertility (Pehlivan-Günaydın et al., 2026; Peng et al., 2024). Meanwhile, as typical oligotrophs, Acidobacteriota are renowned for their capabilities in degrading complex plant residues and participating in iron cycling, which are crucial for the decomposition of recalcitrant organic matter and the maintenance of nutrient turnover in nutrient-limited rhizosphere environments (Ivanova et al., 2020; McReynolds et al., 2025). Pseudomonadota and Acidobacteriota complement each other in ecological niches, with the former responsible for rapid nutrient cycling of labile organic matter and the latter for the decomposition of refractory organic matter, together maintaining the basic structure and functional stability of the rhizosphere microbial community. Actinomycetota, another key member of the shared core microbiome, are key decomposers in soil ecosystems that contribute to the mineralization of recalcitrant organic matter and nutrient cycling (Peng et al., 2024). They represent the major producers of antibiotics, which play critical roles in regulating microbial community structure and suppressing soil-borne pathogens (Nguyen et al., 2023). Additionally, Actinomycetota exhibit strong tolerance to environmental stresses and can promote soil aggregate formation through the production of hyphae and extracellular polysaccharides, thereby improving soil structure and water-holding capacity (Silva-Solar et al., 2024; Xiao et al., 2026). This study found that the relative abundance of Actinomycetota in the rhizosphere soil of infected *S. chinensis* was significantly higher than that in the healthy group. These findings suggest that the rhizosphere microecosystem of infected *S. chinensis* creates a defense barrier to resist pathogen invasion and expansion by enhancing the synthesis and secretion of antibiotics through the enrichment of Actinomycetota communities (Nguyen et al., 2023).

We also found that Acidobacteriota and its subordinate genus *Luteitalea*, as well as *Pseudolabrys* in Pseudomonadota, were the enriched taxa in the healthy group, suggesting that these taxa play crucial roles in maintaining the healthy growth of *S. chinensis*. In contrast, Actinomycetota and its subordinate Gaiellales, *Gemmatimonas* in Gemmatimonadota, and *Bradyrhizobium* in Pseudomonadota were the enriched taxa in the infected group. These taxa show a strong correlation with the diseased state, and their enrichment is likely a result of root rot-induced alterations in the rhizosphere environment, (Huang et al., 2024). Another differential taxon in the infected group is Sphingomonadaceae in Pseudomonadota, with broad-spectrum metabolic capabilities. It can also degrade organic pollutants such as aromatic compounds and polycyclic aromatic hydrocarbons (PAHs) and is more adaptable to harsh rhizosphere environments (Fujita et al., 2020). It also alleviates stresses such as drought, heavy metal stress, and disease incidence (Hafeez et al., 2024). *Bradyrhizobium*, which can form a symbiotic relationship with the host, improves plant nitrogen-use efficiency and stress resistance by synthesizing plant hormones and deaminase (Nemat et al., 2020). Thus, the enrichment of this bacterium in the infected group suggests an adaptive strategy to adversity. Meanwhile, the enrichment of Gaiellales of the Actinomycetota in the infected group accelerates soil carbon loss by decomposing soil organic matter (Wei et al., 2024), consistent with the decreased organic matter content in the infected group. An increase in soil organic matter content effectively inhibits the occurrence of soil-borne diseases (Hao and Ashley, 2021). Studies have found that Gemmatimonadota is widely distributed in various soils, indicating that they are generalist species with diverse metabolic capabilities and can adapt to various nutrients (Mujakić et al., 2022). In this study, the deficiency of nutrients and the reduction of beneficial bacteria in the soil of the infected group led to the enrichment of Gemmatimonadota.

Additionally, we found significant enrichment of Methylomirabilota in the healthy group. Methylomirabilota has independent and efficient methane oxidation capacity (Yao et al., 2024); it exerts a carbon sequestration effect and improves carbon sequestration efficiency. Lan et al. found that the enrichment of Methylomirabilota plays an important role in regulating soil organic carbon storage and soil structure (Lan et al., 2022). Therefore, it can be inferred that Methylomirabilota may have created a favorable rhizosphere environment for the healthy growth of *S. chinensis* by regulating soil carbon cycling and improving the soil microenvironment. Besides, the study’s findings suggested that the significant enrichment of *Luteitalea* in the soil of the healthy group effectively improved the availability of soil phosphorus (Barquero et al., 2024), thereby enhancing soil fertility, consistent with the increased levels of available phosphorus in the soil of the healthy group.

Besides, *Pseudolabrys* was significantly enriched in the healthy group, and its abundance was much higher than that in the infected group. The significantly increased total nitrogen and available nitrogen contents in the soil of the healthy group, along with this enrichment, suggest the role of this genus in soil nitrogen fixation and efficient nitrogen utilization (Wongdee et al., 2018; Yu F. et al., 2022). Further correlation analysis showed that soil hydrolyzable nitrogen content was positively correlated with the abundance of *Pseudolabrys*. The proliferation of *Pseudolabrys* also reconstructs the dominant soil microbial populations, limits the growth of exotic microorganisms, and thus enhances the stability of the soil microbial community (Li et al., 2022).

These results indicate that the differential enrichment of functional genera including *Pseudolabrys* and *Luteitalea* in the rhizosphere of healthy and diseased *S. chinensis* is closely related to the occurrence and development of root rot. Previous studies have demonstrated that *Pseudolabrys* and *Luteitalea* are typical beneficial soil microorganisms (Jing et al., 2026; Yan et al., 2025). The enrichment of these beneficial genera in healthy soil helps maintain the balance of soil nutrient cycling involving nitrogen and phosphorus (Barquero et al., 2024; Yu T. et al., 2022), stabilize the rhizosphere microecosystem and inhibit the colonization and proliferation of pathogens, thus reducing the occurrence of root rot. In contrast, the reduction of these beneficial genera in diseased soil gives rise to disordered nutrient cycling and imbalanced microbial community, which provide favorable conditions for the development of root rot pathogens. Multiple studies have demonstrated that beneficial microorganisms suppress pathogenic microorganisms via multiple synergistic mechanisms. They compete for nutrients and niches through competitive exclusion (Asghar et al., 2024; Prasad et al., 2023), directly inhibit growth via specific antimicrobial compounds (Taylor et al., 2025; Poonguzhali et al., 2025), and enhance rhizosphere colonization by forming biofilms to establish a dominant community (Prasad et al., 2023). In addition, they induce systemic resistance in plants (Asghar et al., 2024; Prasad et al., 2023) and strengthen defense barriers by degrading pathogen cell walls and exerting antagonistic effects (Al-Quwaie, 2024; Alrajeh and Sherif, 2024), thereby reducing pathogen invasion and ultimately controlling plant diseases.

Based on the above results, targeted cultivation and management suggestions are proposed to improve the application value of this study. Culturable beneficial microorganisms including *Pseudolabrys* and *Luteitalea* can be isolated and propagated to develop biological fertilizers for the cultivation of *S. chinensis*, so as to improve soil nitrogen fixation capacity and phosphorus availability. The monitoring of rhizosphere microbial dynamics and soil nutrient status should be strengthened, and functional microbial agents and nutrients including nitrogen and phosphorus should be supplemented timely to maintain the stability of rhizosphere microecosystem. The above measures can be combined with pathogen control during the growth period, providing practical support for the high-quality and sustainable cultivation of *S. chinensis*.

### 4.3 Functional differentiation and metabolic adaptation of rhizosphere bacterial communities in response to root rot infection in *Schisandra chinensis*

The functions of rhizosphere microorganisms are the core embodiment of their adaptation to environmental stress. The present study’s COG functional annotation and differential analysis showed that COG0577 had the highest abundance in both groups, and its abundance in the healthy group was significantly higher than that in the infected group. Combined with LEfSe analysis, it was further clarified that the healthy group was enriched with COG4771, COG0596, and COG1529, while the infected group was only enriched with COG2814. COG0577 is involved in the transport of antimicrobial peptides and is a key functional gene in the plant rhizosphere defense mechanism (Bai et al., 2025b). For instance, the ABC system is known to transport important biomolecules into or out of cells and thereby protect cells from harmful substances (Wang et al., 2019). The decreased abundance of the ABC system leads to reduced transport of antimicrobial substances in the rhizosphere, weakens inhibitory ability against pathogens, and provides conditions for the occurrence of root rot. Similarly, COG4771 (inorganic ion transport and metabolism) is responsible for the transport of siderophores and iron, and it participates in the uptake of certain antimicrobial peptides, playing a key role in bacterial iron metabolism and environmental adaptation (Galperin et al., 2015). Meanwhile, COG0596 (coenzyme transport and metabolism) is involved in the biosynthesis of menaquinone and enhances the tolerance of bacterial communities to oxidative stress (Cimmino et al., 2023). COG1529 (energy production and conversion) plays a key role in aldehyde degradation and exogenous compound detoxification; it also participates in carbon and nitrogen cycles (Steunou et al., 2023; Stipek et al., 1994). The significant enrichment of these three COGs indicated that the functions of rhizosphere bacteria of *S. chinensis* in key physiological and ecological processes (iron metabolism regulation, oxidative stress resistance, detoxification of exogenous harmful substances, and carbon and nitrogen cycling) were enhanced, and the capacities of material transport, energy metabolism, and environmental adaptation of its rhizosphere microecosystem were significantly improved, thereby creating a stable and healthy rhizosphere microenvironment for the growth of *S. chinensis*. Meanwhile, the infected group was enriched with COG2814, associated with carbohydrate transport and metabolism, reflecting a minimalistic survival adaptation strategy under stress.

COG annotation and differential analysis indicated that the differences in rhizosphere bacteria between the healthy and infected groups were mainly concentrated in metabolic functions. Therefore, this study further explored the KEGG metabolic pathways to elucidate the specific biological implications of these metabolic differences. The analysis showed significant differences in the enriched metabolic pathways of rhizosphere bacterial communities between the healthy and infected *S. chinensis*, probably because the soil microorganisms change their metabolic pathways to adapt to the rhizosphere environment (Yu et al., 2023). ABC transporters were the key differential pathway of rhizosphere soil bacteria in infected *S. chinensis*. Generally, ABC transporters assist bacteria in hydrolyzing ATP for nutrient uptake from the environment. The relative enrichment of such protein pathways by the rhizosphere microorganisms is probably related to nutrient deficiency in the rhizosphere of infected *S. chinensis*; this observation suggests that the plant requires more ABC transporters to assist in obtaining nutrients from the soil (Ekiert et al., 2022).

KEGG enrichment analysis of microorganisms in the rhizosphere soil of healthy *S. chinensis* showed that biosynthesis of secondary metabolites, microbial metabolism in diverse environments, and pyruvate metabolism were the major metabolic pathways different between the healthy and infected groups. The production of secondary metabolites helps plants defend against herbivores and pathogen attacks, exert allelopathic effects to regulate the growth of surrounding organisms, and mediate interactions with beneficial microorganisms. The secondary metabolites also act as signal molecules in plant-microbe interactions, precisely regulating the establishment and maintenance of symbiotic relationships between plants and microorganisms (Ketehouli et al., 2025); these changes ensure the healthy growth of *S. chinensis*. The enrichment of microbial metabolism in diverse environments of the healthy group indicates that the microbial community efficiently utilizes diverse substrates and maintains stable functions even when the soil environment fluctuates. Pyruvate is a key intermediate product of glucose metabolism and amino acid metabolism, and its metabolic efficiency determines microbial energy supply and growth rate (Yuan et al., 2022). The active pyruvate metabolism in the healthy rhizosphere indicates vigorous metabolism of the corresponding microbial community.

Further CAZy enzyme annotation revealed core differences in carbohydrate metabolism functions between the two groups. Glycoside hydrolases (GHs, 36.76%) and glycosyl transferases (GTs, 30.97%) were the dominant genes related to carbohydrate metabolism in the rhizosphere soil bacteria of *S. chinensis*, which together constituted the core enzyme system for carbohydrate metabolism. However, significant differences were detected in the enrichment of enzyme families between the healthy group and the infected group. The healthy group mainly enriched a combined enzyme family including GTs, CE, and GHs. GTs catalyze the connection between sugar molecules and other molecules, providing the basis for the synthesis and transfer of monosaccharides and oligosaccharides (Lairson et al., 2008). CE participates in the decomposition of complex carbohydrates and plant cell wall synthesis. The GH family is mainly involved in the decomposition of cellulose and oligosaccharides, responsible for the cleavage of glycoside residues (Drula et al., 2022). The synergistic enrichment of these three enzymes creates a complete carbohydrate metabolic network, which probably accelerates the transformation of soil nutrients, promotes the absorption of nutrients by *S. chinensis* roots, and enhances plant defense capabilities. Specifically, these enzymes participate in cell wall synthesis and maintain the stability of the rhizosphere microecology to enhance plant defense. In addition, the abundances of carbohydrate esterases and polysaccharide lyases in the healthy group were significantly higher than those in the infected group; this rise further enhanced the functions of complex carbohydrate decomposition and root protection (Bhaskar Rao et al., 2020). In contrast, the infected group enriched a single GH family and therefore lacked the synergistic effect of GTs and CE. The core function of the GH family is to hydrolyze glycosidic bonds, and its single enrichment indicates that the carbohydrate metabolism of rhizosphere microorganisms in the infected group is more inclined to decomposition (Iacono et al., 2023). This functional imbalance leads to insufficient synthesis of complex carbohydrates in the rhizosphere soil, limiting the nutrients and support for plant growth and thus inducing root rot incidence.

By integrating the results of soil physicochemical properties, bacterial community, and functional analyses, we elucidated the microecological response induced by pathogen colonization in the rhizosphere of *S. chinensis*. Specifically, pathogen colonization induces rhizosphere soil acidification, disordered organic matter decomposition, and decreased available nutrients (HN and AK), thereby forming an adverse microenvironment (Spooren et al., 2024). The adverse environment inhibits the growth of healthy core taxa such as Acidobacteriota through nutrient screening and pH selection, while promoting the enrichment of opportunistic taxa such as Actinomycetota and Gemmatimonadota; these changes result in community structure disorder (Zhu et al., 2023). The decline in core taxa directly leads to the collapse of their associated functional networks (Ossowicki et al., 2021). The defense-coordination function of the healthy group, characterized by “coordinated and efficient metabolism (COG)-secondary metabolite synthesis (KEGG)-complex carbon metabolism (CAZy multi-enzyme system)”, is lost. Instead, the infected group exhibits simplified survival functions, including “single metabolism (COG2814)-ABC transporters (KEGG)-glycoside hydrolase enrichment (CAZy GH family)” (Low et al., 2023; Zhang T. et al., 2023). Soil functional degradation leads to the loss of the rhizosphere’s ability to inhibit pathogens, and further expansion of pathogens exacerbates the deterioration of soil physicochemical properties (Chaudhary et al., 2024).

## 5 Conclusion

The present study found that the occurrence of root rot in *S. chinensis* is closely associated with an imbalance in the rhizosphere environment-bacterial community-function system. We identified Acidobacteriota, its subordinate taxa, *Pseudolabrys* of Pseudomonadota, and Candidatus_Methylomirabilota as the core bacterial biomarkers in the rhizosphere of healthy *S. chinensis*. Besides, a functional degradation of rhizosphere bacteria was detected during root rot incidence, with the bacterial metabolic network shifting from a complex synergistic network in healthy rhizospheres to a simple survival network in infected rhizospheres. HN and AK were identified as the key nutrients regulating the structure and function of the rhizosphere bacterial community.

Future research should focus on exploring the specific mechanisms by which different regulatory measures, including various pH adjustment methods, N and K fertilizer ratio adjustments, and functional bacterial screening, modulate the microecosystem of *S. chinensis* rhizosphere. Additionally, the potential functional shifts identified in this study should be validated via metatranscriptomic or metaproteomic approaches. These studies will provide a robust empirical basis for constructing an efficient and precise prevention and control strategy, facilitating the sustainable and high-quality development of the *S. chinensis* industry.

## Data Availability

The metagenomic data in this study are available in the NCBI database, deposited in the NCBI BioProject platform, with the accession number PRJNA1394726. The accession numbers corresponding to each sample sequence are SRR36612685 - SRR36612690.

## Footnotes

1
http://hmmer.janelia.org/search/hmmscan

2
http://www.cazy.org/

## Supplementary material

Supplementary Figure S1 The map of study site.

Supplementary Figure S2 Sampling schematic diagram showing the field layout and five-point sampling strategy.

Supplementary Figure S3 Rarefaction curves based on the Shannon index for all rhizosphere soil samples. C—infected group; D—healthy group.

Supplementary Figure S4 Bacterial phylum level composition of rhizosphere soils from healthy and infected *Schisandra chinensis*. JK—healthy group; BZ—infected group.

Supplementary Figure S5 Bacterial genus level composition of rhizosphere soils from healthy and infected *Schisandra chinensis*. JK—healthy group; BZ—infected group.

Supplementary Figure S6 COG functional classification of rhizosphere bacteria from healthy and infected *Schisandra chinensis*. JK—healthy group; BZ—infected group.

Supplementary Figure S7 LEfSe analysis of COG functional profiles of rhizosphere bacteria from healthy and infected *Schisandra chinensis*. JK—healthy group; BZ—infected group.

Supplementary Figure S8 Pearson correlation analysis.

Supplementary Table S1 Variance inflation factors (VIF) of rhizosphere soil nutrient indicators in *Schisandra chinensis*.
