# Supplementary figures and images for: Metagenomic sequencing reveals structural and functional differentiation of rhizosphere bacterial communities driven by nitrogen and potassium deficiency associated with root rot of Schisandra chinensis
Source: Front Microbiol. 2026 May 13;17:1827096. doi: 10.3389/fmicb.2026.1827096 (PMC13212081; doi:10.3389/fmicb.2026.1827096)

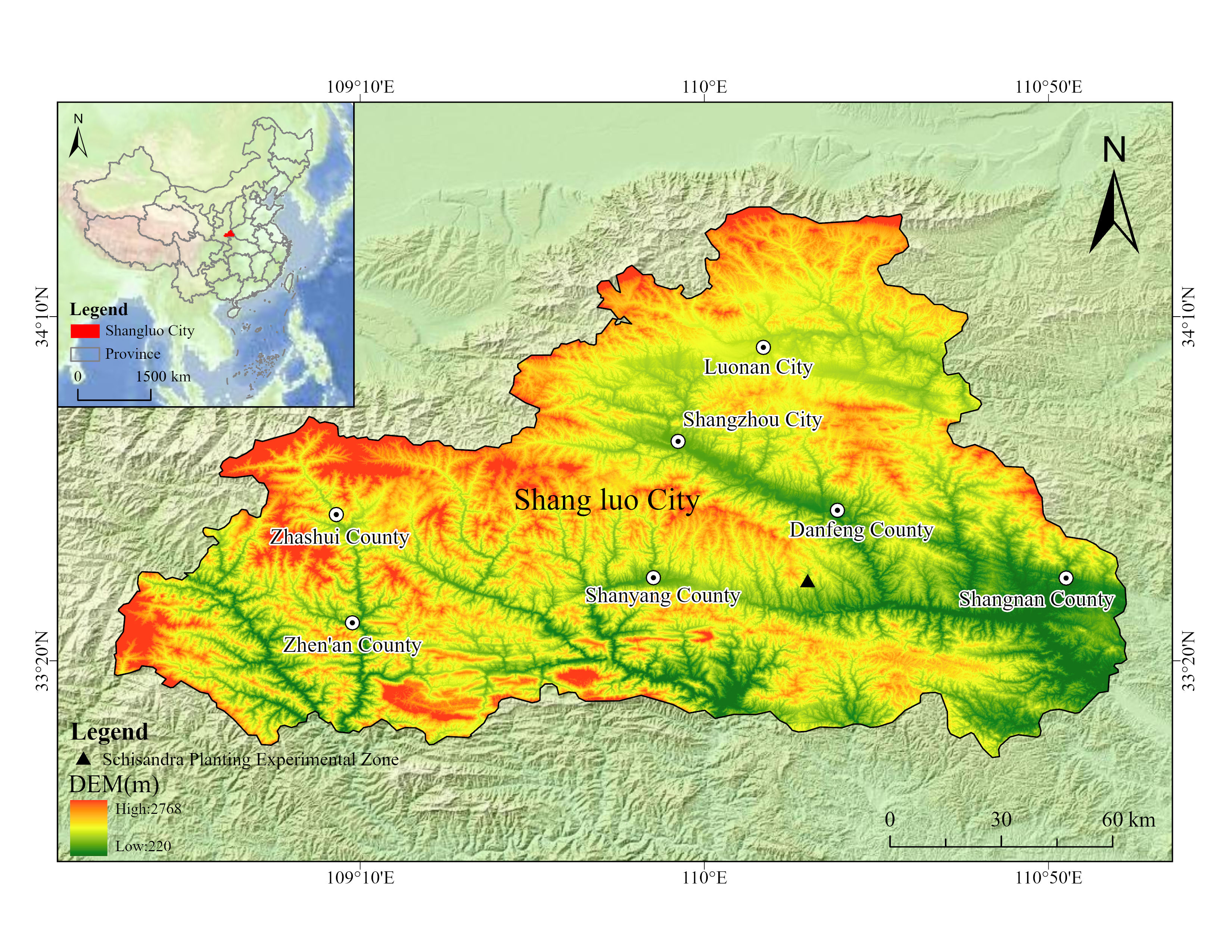

Supplement: Supplementary Figure S1 — The map of study site. [file Image_1.JPEG]

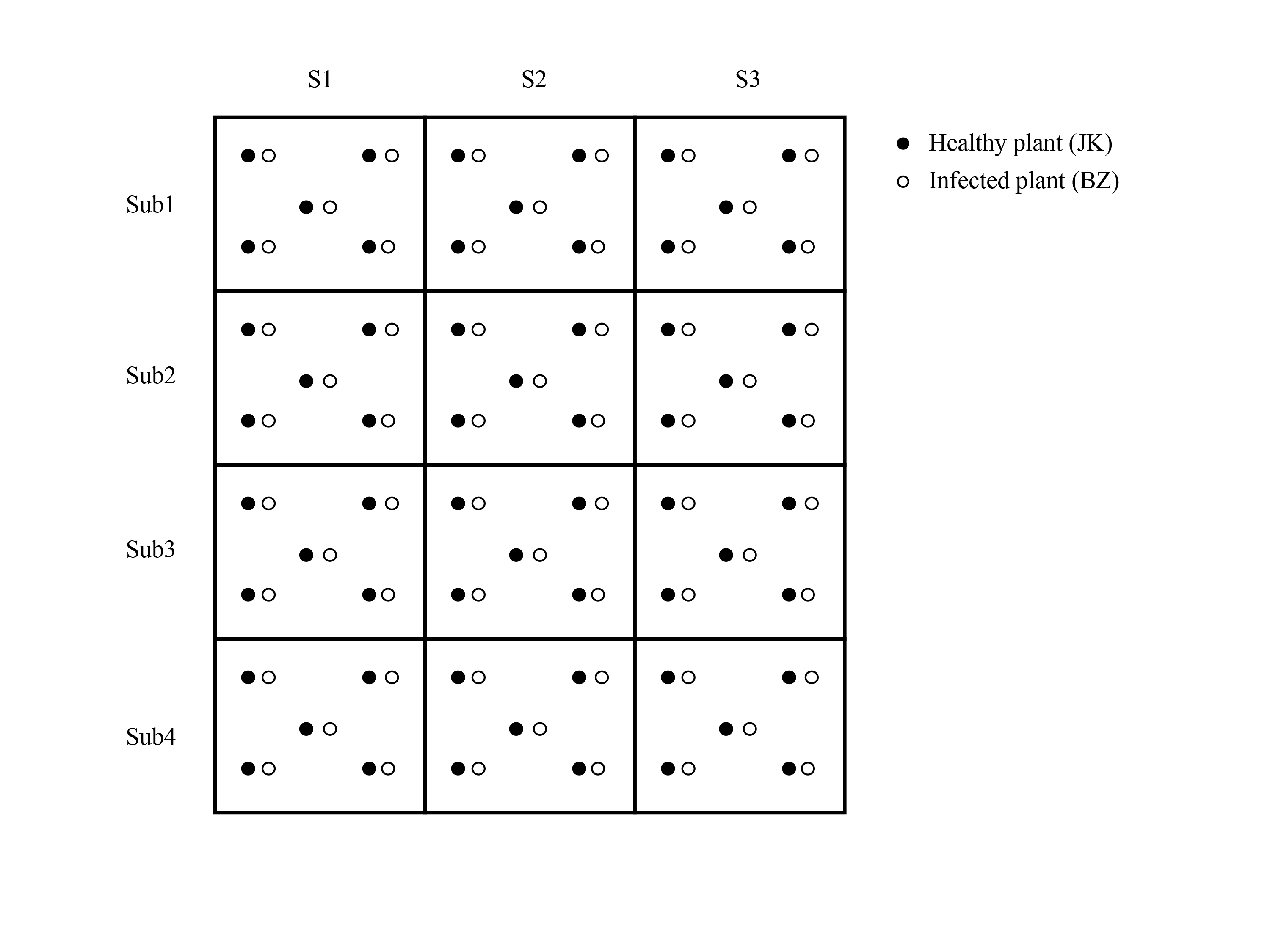

Supplement: Supplementary Figure S2 — Sampling schematic diagram showing the field layout and five-point sampling strategy. [file Image_2.TIF]

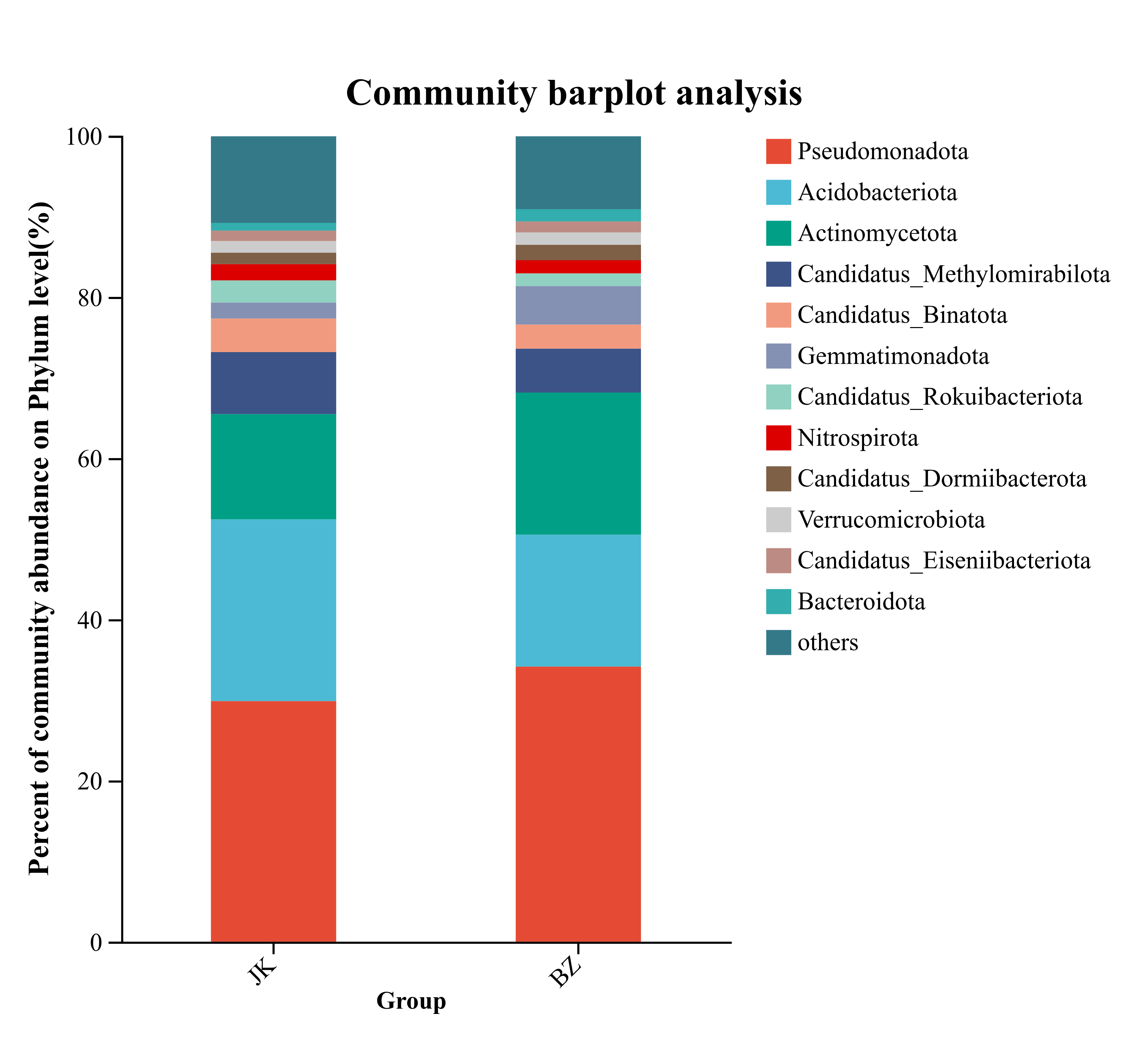

Supplement: Supplementary Figure S4 — Bacterial phylum level composition of rhizosphere soils from healthy and infected Schisandra chinensis. JK—healthy group; BZ—infected group. [file Image_4.TIF]

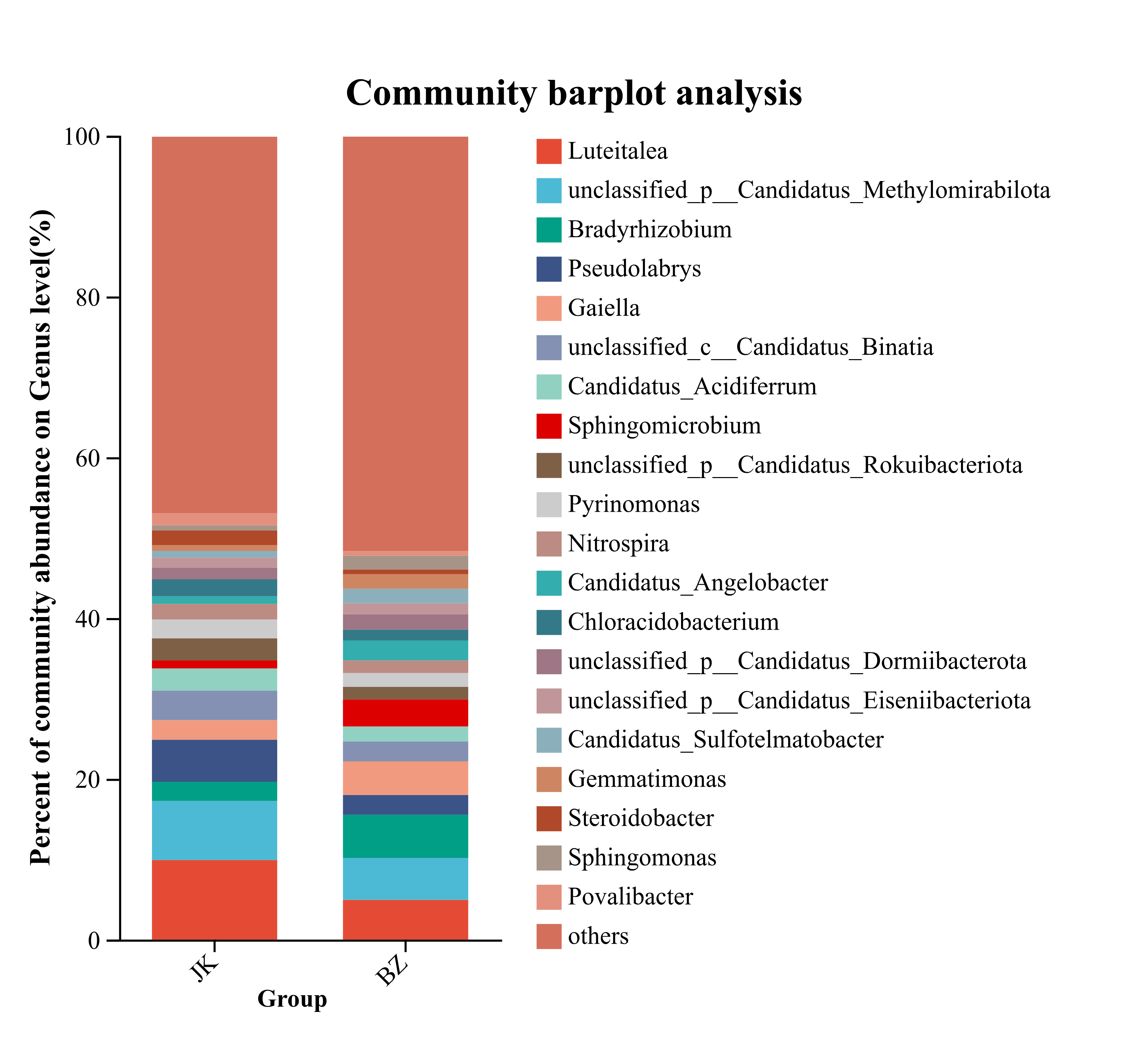

Supplement: Supplementary Figure S5 — Bacterial genus level composition of rhizosphere soils from healthy and infected Schisandra chinensis. JK—healthy group; BZ—infected group. [file Image_5.TIF]

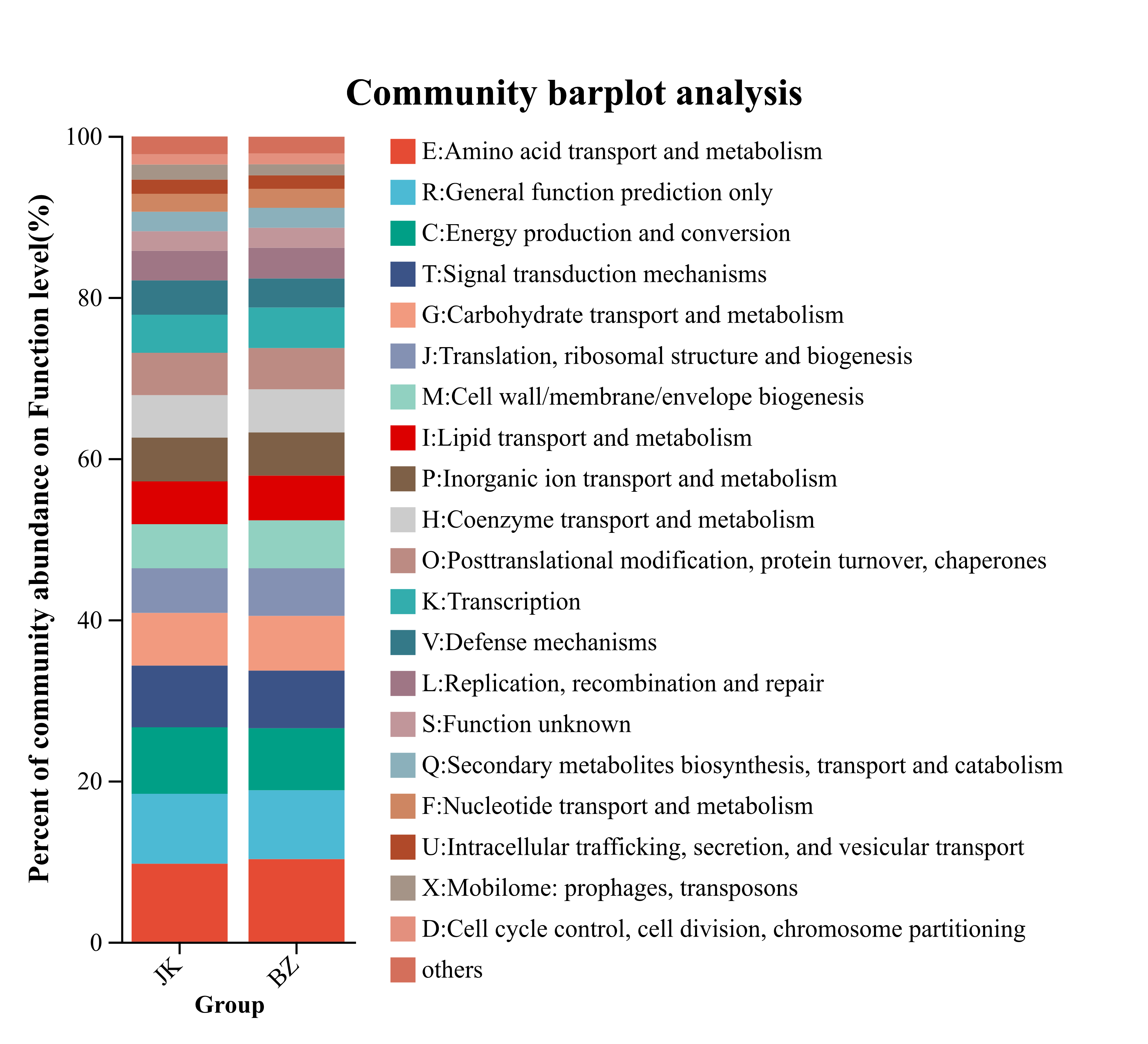

Supplement: Supplementary Figure S6 — COG functional classification of rhizosphere bacteria from healthy and infected Schisandra chinensis. JK—healthy group; BZ—infected group. [file Image_6.TIF]

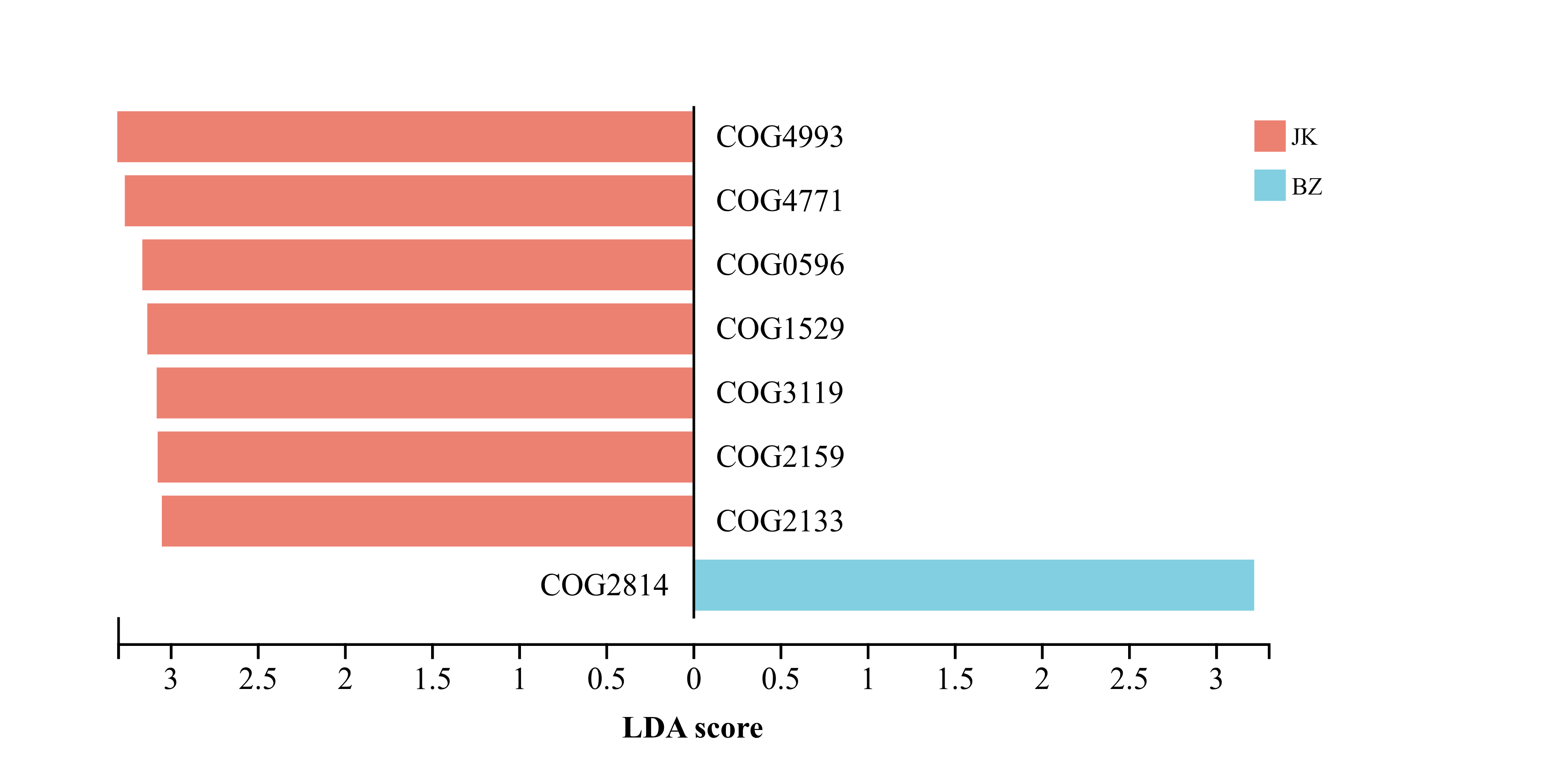

Supplement: Supplementary Figure S7 — LEfSe analysis of COG functional profiles of rhizosphere bacteria from healthy and infected Schisandra chinensis. JK—healthy group; BZ—infected group. [file Image_7.TIF]

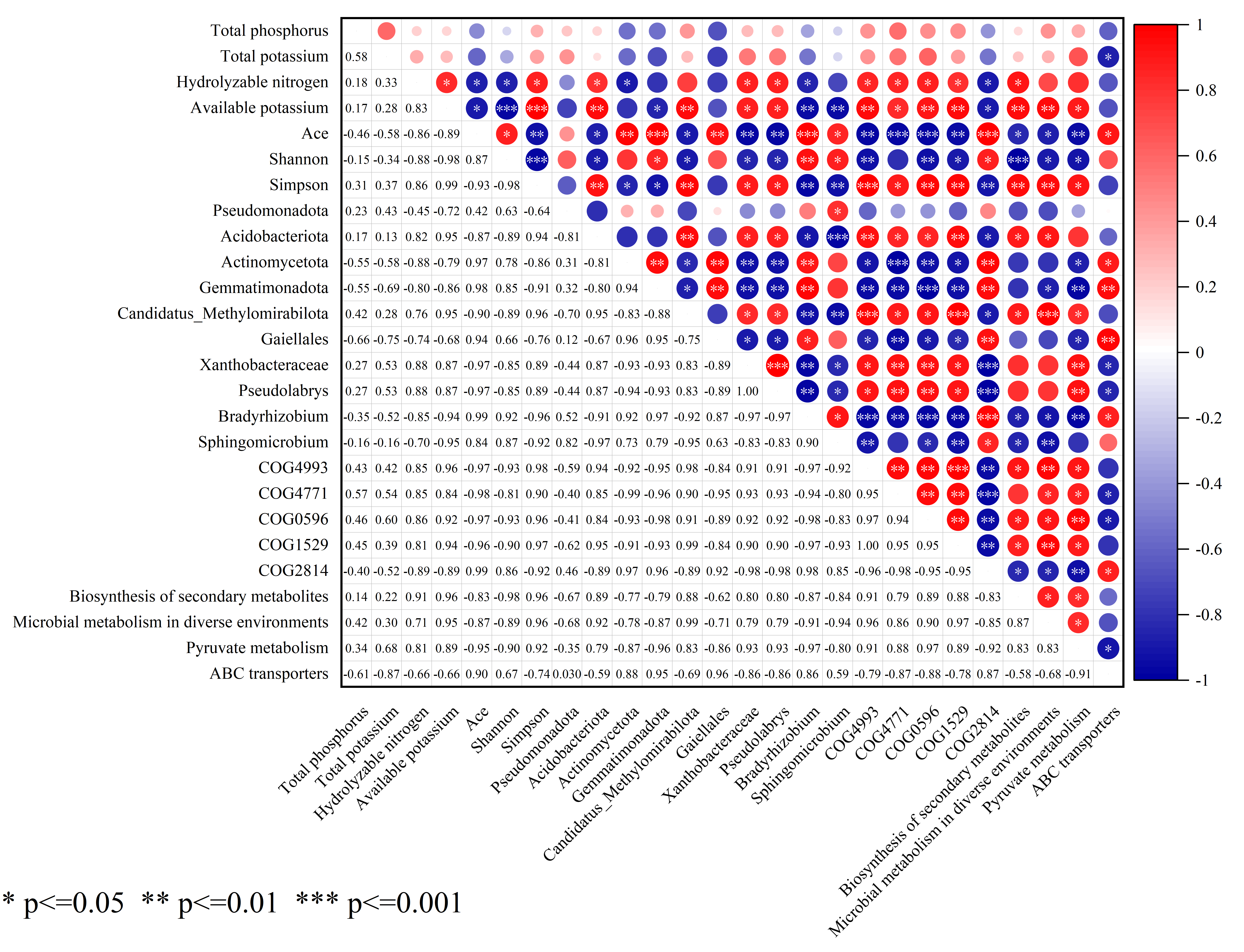

Supplement: Supplementary Figure S8 — Pearson correlation analysis. [file Image_8.TIF]
