# Supplementary material for: Metagenomic sequencing reveals structural and functional differentiation of rhizosphere bacterial communities driven by nitrogen and potassium deficiency associated with root rot of Schisandra chinensis
Source: Front Microbiol. 2026 May 13;17:1827096. doi: 10.3389/fmicb.2026.1827096 (PMC13212081; doi:10.3389/fmicb.2026.1827096)
Supplement: Supplementary Table S1 — Variance inflation factors (VIF) of rhizosphere soil nutrient indicators in Schisandra chinensis. [file Table_1.DOCX]

Table S1. Variance inflation factors (VIF) of rhizosphere soil nutrient indicators in *Schisandra chinensis*

|  | Total phosphorus | Total potassium | hydrolyzable nitrogen | Available potassium |
| --- | --- | --- | --- | --- |
| Variance inflation factor | 1.53 | 1.68 | 3.53 | 3.35 |
